# Catastrophic health expenditure during the COVID-19 pandemic in five countries: a time-series analysis

**DOI:** 10.1016/S2214-109X(23)00330-3

**Published:** 2023-09-19

**Authors:** Annie Haakenstad, Corinne Bintz, Megan Knight, Kelly Bienhoff, Horacio Chacon-Torrico, Walter H Curioso, Joseph L Dieleman, Anna Gage, Emmanuela Gakidou, Simon I Hay, Nathaniel J Henry, Akram Hernández-Vásquez, Judith S Méndez Méndez, Héctor J Villarreal, Rafael Lozano

**Affiliations:** a Institute for Health Metrics and Evaluation, University of Washington, Seattle, WA, USA; b Department of Health Metrics Sciences, University of Washington, Seattle, WA, USA; c Department of Biomedical Informatics and Medical Education, University of Washington, Seattle, WA, USA; d Faculty of Health Sciences, Southern Scientific University, Lima, Peru; e Vice Rectorate for Research, Continental University, Lima, Peru; f Center of Excellence in Economic and Social Research in Health, San Ignacio de Loyola University, Lima, Peru; g School of Government and Public Transformation, Monterrey Institute of Technology, Mexico City, Mexico; h School of Medicine, National Autonomous University of Mexico, Mexico City, Mexico

## Abstract

**Background:**

The COVID-19 pandemic disrupted health systems in 2020, but it is unclear how financial hardship due to out-of-pocket (OOP) health-care costs was affected. We analysed catastrophic health expenditure (CHE) in 2020 in five countries with available household expenditure data: Belarus, Mexico, Peru, Russia, and Viet Nam. In Mexico and Peru, we also conducted an analysis of drivers of change in CHE in 2020 using publicly available data.

**Methods:**

In this time-series analysis, we defined CHE as when OOP health-care spending exceeds 10% of consumption expenditure. Data for 2004–20 were obtained from individual and household level survey microdata (available for Mexico and Peru only), and tabulated data from the National Statistical Committee of Belarus and the World Bank Health Equity and Financial Protection Indicator database (for Viet Nam and Russia). We compared 2020 CHE with the CHE predicted from historical trends using an ensemble model. This method was also used to assess drivers of CHE: insurance coverage, OOP expenditure, and consumption expenditure. Interrupted time-series analysis was used to investigate the role of stay-at-home orders in March, 2020 in changes in health-care use and sector (ie, private *vs* public).

**Findings:**

In Mexico, CHE increased to 5·6% (95% uncertainty interval [UI] 5·1–6·2) in 2020, higher than predicted (3·2%, 2·5–4·0). In Belarus, CHE was 13·5% (11·8–15·2) in 2020, also higher than predicted (9·7%, 7·7–11·3). CHE was not different than predicted by past trends in Russia, Peru, and Viet Nam. Between March and April, 2020, health-care visits dropped by 4·6 (2·6–6·5) percentage points in Mexico and by 48·3 (40·6–56·0) percentage points in Peru, and the private share of health-care visits increased by 7·3 (4·3–10·3) percentage points in Mexico and by 20·7 (17·3–24·0) percentage points in Peru.

**Interpretation:**

In three of the five countries studied, health systems either did not protect people from the financial risks of health care or did not maintain health-care access in 2020, an indication of health systems failing to maintain basic functions. If the 2020 response to the COVID-19 pandemic accelerated shifts to private health-care use, policies to cover costs in that sector or motivate patients to return to the public sector are needed to maintain financial risk protection.

**Funding:**

The Bill & Melinda Gates Foundation.

## Introduction

The COVID-19 pandemic was a shock to health systems worldwide, testing their ability to maintain basic functions and adapt swiftly.^1^ It is important to understand how well health systems protected households from financial hardship due to health-care costs in this time of crisis. Financial protection is a core element of universal health coverage (UHC) and was included in the Sustainable Development Goal (SDG) 3.8.2 on UHC.^2^

Initial analyses of the COVID-19 pandemic's impact underscored reductions in households’ capacity to pay for health care—the denominator of catastrophic health expenditure (CHE).^3^ Global gross domestic product (GDP) decreased by 3·2%,^4^ extreme poverty increased by 9·3%,^5^ and more than 250 million jobs were lost in 2020.^6^, ^7^ In the short term, decreases in the capacity to pay would result in increases in CHE if out-of-pocket (OOP) expenditure held steady in 2020. In the long term, the COVID-19 pandemic could have implications for government revenues and the solvency of insurance programmes, which could weaken financial protection.

However, the numerator of CHE, OOP expenditure, also probably changed during the COVID-19 pandemic. On one hand, OOP spending could have risen. In countries where COVID-19 care was not subsidised, the costs of COVID-19 testing and care could have added to pre-existing OOP spending.^8^ OOP spending could also have risen because more patients with COVID-19 used the private sector (in which OOP costs are higher) to avoid exposure to COVID-19 in the public sector.^9^, ^10^ Alternatively, OOP spending could have dropped. Reductions in the use of non-COVID-19 care could have offset the use of COVID-19 care.^11^ Stay-at-home orders and fear of exposure to COVID-19 could have kept potential users away from health facilities, driving up rates of forgone care.^12^ Shortages of health-care workers and personal protective equipment and other capacity constraints limited the ability of health systems to maintain essential services.^13^, ^14^, ^15^, ^16^ Administrative data have shown that millions fewer vaccinations occurred in 2020 as compared with 2019, and disruptions to other maternal and child health services in 2020.^17^, ^18^, ^19^

**Research in context**

**Evidence before this study**

We conducted PubMed, MedRxiv, and Google searches of “Catastrophic health expenditure and COVID-19” and “OOP and COVID-19” from database inception to July 6, 2023. Although numerous commentaries and viewpoints have been published, only WHO and the World Bank's Global Monitoring Report on Financial Protection in Health 2021 used household data to estimate catastrophic health expenditure (CHE) empirically and reported national-level CHE for Belarus, Russia, and Viet Nam. In peer-reviewed journals, we found 25 studies that examined out-of-pocket (OOP) payments or catastrophic health expenditure for COVID-19 testing, treatment, and hospitalisation, and one study that looked at CHE comprehensively in one country (Iran) without comparing with historical data. No existing peer-reviewed studies have examined how OOP spending compared with consumption expenditure or income in 2020 relative to historical patterns and the underlying causes of change across countries. Tracking of broader indicators of economic and social wellbeing (such as income, employment, and poverty) by the International Monetary Fund, World Bank, International Labour Organization, and others showed large reductions in capacity to pay for health care in 2020.

**Added value of this study**

This study assessed financial hardship due to health-care costs and health-care use across countries during the COVID-19 pandemic in depth for the first time. We examined trends in CHE and investigated its drivers. We used all 2020 household expenditure data in the public domain by the end of 2022 that included health expenditure to track CHE over time in Belarus, Mexico, Peru, Russia, and Viet Nam in 2020. The five countries studied have distinct health systems and COVID-19 experiences, providing diverse contexts in which to analyse financial hardship during the COVID-19 pandemic. Additionally, our study delves into the mechanisms underlying change in CHE in 2020. We assessed the effect of stay-at-home orders on health-care use and sector (private *vs* public) with an interrupted time-series analysis.

**Implications of all the available evidence**

CHE did not universally increase during the pandemic—just two of the five countries studied (Mexico and Belarus) had increases higher than expected based on pre-2020 trends. However, decreases in health-care use underpinned steady CHE rates in at least two of the five countries (Peru and Russia), indicating the same financial hardship occurred with less health care received. Local factors were important, including the discontinuation of Seguro Popular in Mexico and the large drop in mobility due to stay-at-home orders in Peru. In Mexico and Peru, the private sector showed capacity to sustain higher patient volumes than other health-care providers, an indication of the private sector's contribution to the resilience of health systems in 2020. However, if the pandemic accelerated a longer term shift to private-sector care, policies geared towards improving financial coverage or incentivising patients to return to the public sector will be required to maintain financial risk protection.

To date, no peer-reviewed research has investigated in depth the impact of the COVID-19 pandemic on financial risk protection across multiple countries. WHO and the World Bank's Global Monitoring Report on Financial Protection in Health depicted 2020 CHE over time for five anonymous countries, but did not indicate which countries were shown,^19^ contributing to knowledge about 2020 CHE but prohibiting discussion of the connection between CHE, the severity of the COVID-19 pandemic, and features of health-care systems. Other studies have looked at single countries, examined COVID-19 care spending, or only assessed OOP spending (but not CHE).^4^, ^6^, ^7^, ^11^, ^20^, ^21^, ^22^, ^23^, ^24^, ^25^, ^26^, ^27^, ^28^, ^29^, ^30^, ^31^, ^32^, ^33^, ^34^, ^35^, ^36^, ^37^, ^38^, ^39^, ^40^, ^41^, ^42^, ^43^ The absence of cross-country comparison of 2020 CHE limits understanding of health system resilience and the adaptation of policies to address financial hardship going forward.

We fill this gap in the literature by assessing trends in CHE across all countries for which 2020 household expenditure data were published by the end of 2022: Belarus, Mexico, Peru, Russia, and Viet Nam. We compare 2020 rates of CHE and its drivers with historical trends, and analyse whether they are linked with stay-at-home orders. Our results shed light on the resilience of financial protection across countries during the COVID-19 pandemic and identify the contributing factors that policy makers should consider to ensure financial protection is strong going forward.

## Methods

### Study design

In this time-series analysis, we focused on assessing change in CHE and its drivers in 2020, using data aggregated to the national level and year. The following drivers of CHE were assessed: consumption expenditure, OOP expenditure, health-care use, insurance coverage, and sector of health-care use (public *vs* private). CHE was assessed in the only five countries worldwide with data publicly available by the end of 2022 for CHE in 2020 and in the preceding 2004–19 period. Drivers of CHE were assessed in the only two countries with publicly available data on both CHE and its drivers: Mexico and Peru. We constructed a counterfactual scenario predicting CHE and its drivers if they had continued their trajectory before the COVID-19 pandemic. We compared these predictions with the observed values in all five countries to determine whether 2020 rates were different to the rates predicted by historical trends. In Peru and Mexico, we also examined whether changes in CHE could be explained by how health-care use and sector changed following the initial lockdowns in March, 2020, using an interrupted time-series design and data aggregated to the national level and month.

This study complies with the Guidelines for Accurate and Transparent Health Estimates Reporting statement, with further information provided in the appendix (pp 6–7). Because we use only secondary, de-identified data, ethical approval was not required for this study.

### Data sources and collection

Our analysis focused on population-level estimates of CHE and its drivers in five countries: Belarus, Mexico, Peru, Russia, and Viet Nam. The data sources are described in the appendix (p 8). We were unable to obtain microdata for Belarus, Russia, and Viet Nam, so we used national-level CHE estimates reported by the National Statistical Committee of the Republic of Belarus and the Global Monitoring Report on Financial Protection in Health published by the World Bank and WHO in 2021.^19^ More detailed data were available for Mexico and Peru. Disruptions to data collection were minimal in Belarus and Viet Nam because physical distancing orders were limited in these two countries. In Mexico, the National Survey of Household Income and Expenses^44^ was conducted in 2020 with the same methods (face-to-face interviews) and time of year (August–November) as in previous iterations and avoided the initial lockdown period. In Peru, the National Household Survey on Living Conditions and Poverty^45^ was conducted face to face between January and March, 2020, and October and December, 2020, but between March 16 and Sept 30, 2020, telephone interviews and other alternative methods were used to collect data. Despite these changes, respondent characteristics in the 2020 surveys were similar to previous years (appendix pp 9–10).

### Procedures and outcomes

CHE was defined as when household OOP spending exceeded 10% (or 25% as an alternative threshold) of household consumption expenditure, consistent with the definition in SDG 3.8.2.^2^ OOP and consumption expenditure reported for different recall periods were scaled linearly to the same period. Population-level CHE was defined as the share of all households incurring CHE.

Other CHE drivers were also based on self-report. Respondents identified whether household members were covered by insurance, used health care, and where the health care was obtained (eg, at a public hospital, public clinic, private clinic, or pharmacy)**.** In the surveys from Mexico and Peru, sex was self-reported and collected as a binary indicator (ie, male and female only). In Mexico, respondents reported when and where health-care use took place for an illness or accident in the year before the survey. Health-care use rates were based on counts of health-care use per month divided by the total number of respondents in the survey. In Peru, respondents reported whether and where care was used in the past 4 weeks (the survey was conducted in every month of 2020) for an illness or accident. Health-care use rates were based on the counts of health-care use per month divided by the number of survey respondents in that month in Peru. These differences in health-care use definition, recall period, and population make health-care use rates in the two countries not directly comparable. We categorised visits in private clinics, hospitals, and pharmacies in Mexico as taking place in the private sector. In Peru, the private sector was defined as private consultations, clinics, and pharmacies. Written or verbal informed consent from survey respondents was obtained from the respective institutions that implemented data collection.^45^, ^46^, ^47^, ^48^, ^49^, ^50^

### Statistical analysis

First, we assessed whether 2020 CHE rates and drivers were different from what was expected based on pre-2020 trends using a time-series-based forecasting approach. We derived our approach from the forecasting method used in the Global Burden of Diseases, Injuries, and Risk Factors Study (GBD).^51^ Because of the short time frame (retrospectively and prospectively) and small number of countries, we focused on the GBD latent trends approach. We assembled four models with autoregressive terms only: level regressed on lag-level and difference regressed on lag-difference, each with and without a constant. Each country has a different number of datapoints for 2004–19, the period used to estimate coefficients and model performance. Russia has the fewest years of data (2004, 2010–14, and 2020) and Belarus has the most (2004–20). Models with multiple lags and moving averages were ruled out because of the limited years of data and their omission in the GBD latent trends approach. We stratified models by country. We compared the performance of the models with out-of-sample root mean squared error. Combining four models in an ensemble out-performed any single model for CHE (appendix pp 13–20).

We compared the predictions with the observed 2020 points. Uncertainty in the predictions was estimated by applying 1000 draws capturing uncertainty from the four models to 1000 draws capturing the uncertainty of the data (generated using standard errors accounting for the sample size and sampling design in the aggregated country-year values), and taking the mean, 2·5th, and 97·5th percentiles of the draws. We considered the 2020 observed points to be different than the predicted 2020 values if the uncertainty intervals (UIs) did not overlap.

Second, we assessed how health-care use changed when stay-at-home orders were put in place on March 15, 2020, in Peru and March 31, 2020, in Mexico, focusing on the two measures that can be estimated by month: health-care visits per person and the share of health-care visits that took place in the private sector. These data were aggregated by country and month of each year. We first visualised time trends. Monthly 2020 health-care use rates are depicted relative to monthly health-care rates in 2019 in Peru and 2018 in Mexico (the most recent year of data) to net out seasonal patterns. No seasonal trends were apparent in the private-sector share of health care, so it is depicted by month starting in January, 2019 in Peru and January, 2018 in Mexico. In Mexico, we extrapolated the 2018 linear trend in private-sector share to 2019 to assess whether January, 2020 rates corresponded with what would be expected based on the 2018 trend.

We used an interrupted time-series approach to test for a shift in rates and trends in March, 2020. The dependent variables were in the same units as the visualisations. We estimated the following ordinary least squares regression:

$$Y_{t}=\beta_{1}+\beta_{2}month+\beta_{3}I(COVID19)+\beta_{4}I(COVID19)*month+{ɛ}_{t}$$

Where *Y*_t_ represents relative health-care use or private-sector share in 2018, 2019, or 2020 by month; month is a count for month; *I*(*COVID-19*) is an indicator representing April to December, 2020; and *I*(*COVID-19*) × month is an interaction between the COVID-19 period and the month count. Our quantities of interest are β_3_, the change in the outcome, and β_4_, the shift in monthly trends, when stay-at-home orders commenced. In Mexico, data were not collected for 2019 (the surveys are run every other year), and thus we added an additional year intercept for 2020 in the private share regression, to ensure our estimates do not capture the secular trend between the end of 2018 and the beginning of 2020. The error term in the regression, ɛ_t_, was calculated as Newey-West standard errors to address serial correlation.

In all our analyses, we used the survey weights provided by statistical agencies to ensure our results are nationally representative. Analyses and figures were generated with R (version 4.2.1).

### Role of the funding source

The funder of the study had no role in study design, data collection, data analysis, data interpretation, or writing of the report.

## Results

**Figure 1:**
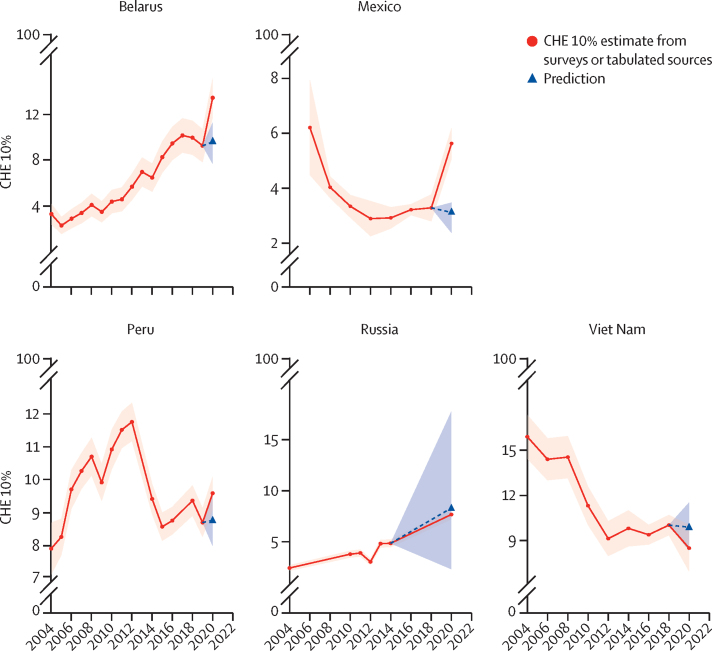
CHE trends in Belarus, Mexico, Peru, Russia, and Viet Nam, 2004–20 CHE was defined as when out-of-pocket health-care spending exceeds 10% of consumption expenditure and the graphs depict the proportion of households with CHE (10%). The orange shaded area represents the uncertainty surrounding the mean (red datapoints) based on the data. The blue dotted line and arrowhead represents the mean prediction for 2020 based on past trends in CHE. The blue shaded area represents the uncertainty surrounding the prediction. CHE=catastrophic health expenditure.

Changes in CHE in 2020 differed substantially depending on the country (figure 1). Increases in CHE occurred in Belarus (13·5% [95% UI 11·8–15·2] in 2020 *vs* the predicted 9·7% [7·7–11·3]) and in Mexico (5·6% [5·1–6·2] in 2020 *vs* the predicted 3·2% [2·5–4·0]). In Russia, Peru, and Viet Nam, predicted CHE rates were similar to the observed CHE rates in 2020 (appendix pp 18–19).

**Figure 2:**
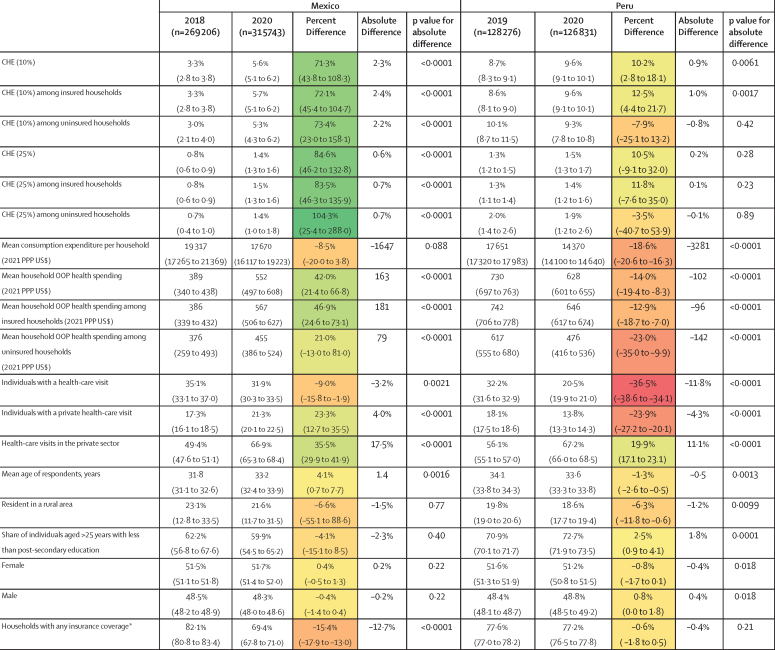
Economic and social household indicators in 2020 versus 2018 (Mexico) and 2019 (Peru) Data are percentage (95% uncertainty interval [UI]), mean (95% UI), or absolute percentage point difference, unless otherwise specified. Values based on the respective surveys, weighted to be nationally representative using the survey weights provided by the respective data collection institutions. Definition of health-care visits differed in Peru and Mexico. In Mexico, the recall period for health-care visits was the past year, so the denominator is all respondents. In Peru, the recall period for visits was the past 4 weeks, so the denominator is all respondents asked to recall visits in the month of reference. CHE=catastrophic health expenditure. OOP=out of pocket. PPP=purchasing power parity. *Defined as households in which any household member is insured; this definition results in different coverage estimates when compared with calculating insurance coverage among individuals.

Figure 2 presents basic information about Mexico and Peru in 2020 versus the most recent previous year of survey data (2018 in Mexico and 2019 in Peru). In Mexico, CHE (defined by the 10% threshold) increased by more than 70% in 2020 relative to 2018 overall, with CHE among insured (5·7%, 95% UI 5·1 to 6·2) and uninsured (5·3%, 4·3 to 6·2) households increasing at a similar rate as compared with 2018 (2·4 percentage points [by 72·1%, 45·5 to 104·7] for insured people, and 2·2 percentage points [by 73·4%, 23·0 to 158·1] for uninsured people). Peru had a more modest increase of 0·9 percentage points in CHE (defined by the 10% threshold), with CHE rates among uninsured individuals dropping by 0·8 percentage points. In Mexico, the share of households with health insurance decreased by 12·7 percentage points**,** whereas there was only a small decrease in Peru (–0·4%). Consumption expenditure dropped more notably in Peru (–18·6%, –20·6 to –16·3) than in Mexico (–8·5%, –20·0 to 3·8). In Mexico, OOP spending increased substantially (42·0%, 21·4 to 66·8), whereas OOP expenditure dropped in Peru (–14·0%, –19·4 to –8·3). Health-care use decreased in both countries in 2020, but the use of the private sector increased in Mexico overall. A larger share of visits took place in the private sector in 2020 versus the comparison years in both countries, although in Peru the private sector counts declined along with health-care use overall. There were minimal differences in basic sociodemographics between the two years of data in each country.

**Figure 3:**
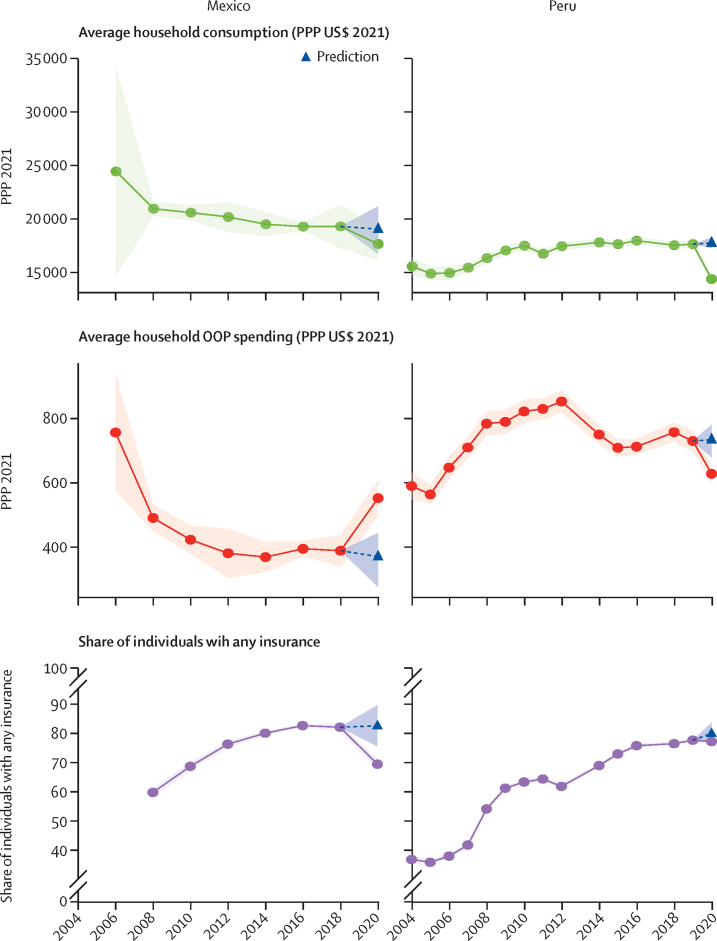
Drivers of CHE in Mexico and Peru, 2004–20 The green, orange, and purple shaded areas represent the uncertainty surrounding the mean (represented by green, red, and purple datapoints) based on the data. The blue dotted lines and arrowhead represent the mean prediction for 2020 based on past trends in CHE. The blue shaded area represents the uncertainty surrounding the prediction. CHE=catastrophic health expenditure. OOP=out-of-pocket. PPP=purchasing power parity.

Figure 3 shows changes in drivers of CHE relative to the 2020 predictions. In Mexico, insurance rates were lower (69·4%, 95% UI 67·8–71·0) than the expected value (82·6%, 75·6–89·6), whereas OOP health spending (552 purchasing power parity US dollars [PPP] 2021, 497–608) was higher than expected (370 PPP, 277–444); consumption expenditure was not different than expected. By contrast, in Peru, consumption expenditure (14 370 PPP, 14 100–14 640) was lower than expected (17 763 PPP, 17 292–18 263), as was OOP health spending (628 PPP, 601–655 *vs* 773 PPP, 679–780), whereas insurance rates were not different than expected.

**Figure 4:**
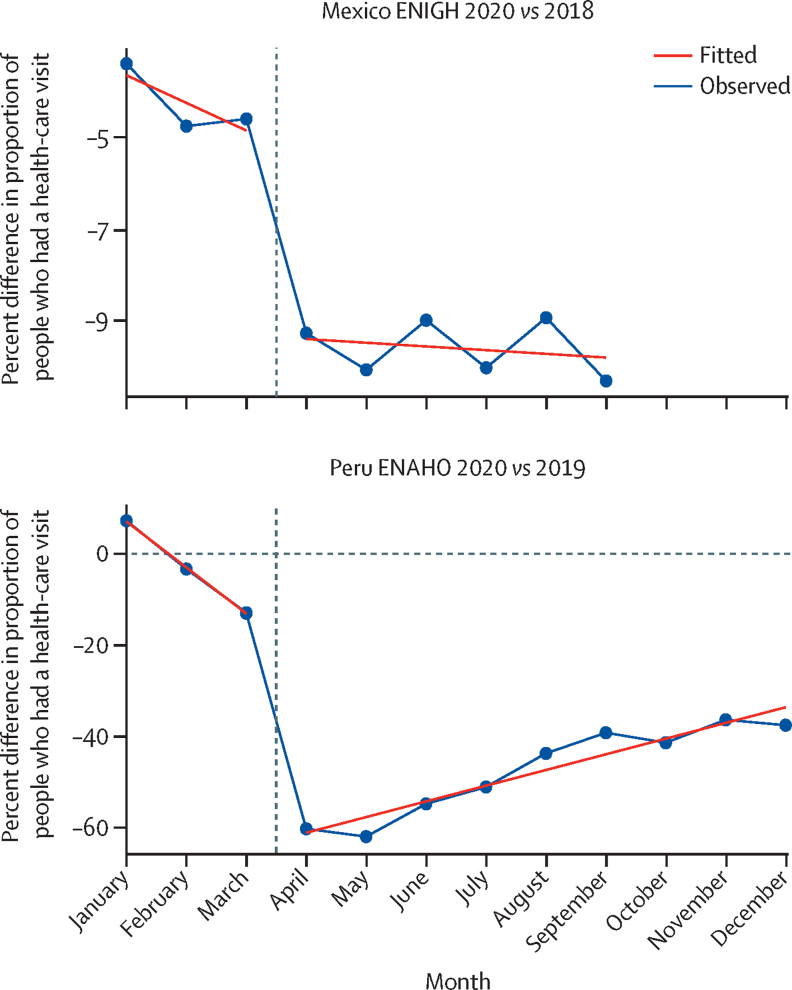
Health-care visits per person in 2020 versus 2018 (Mexico) and 2019 (Peru) The blue line connects the observed datapoints. The red line represents the linear fit of the data. ENIGH=Encuesta Nacional de Ingresos y Gastos de los Hogares. ENAHO=La Encuesta Nacional de Hogares.

Figure 4 depicts monthly changes in use of health care in Peru and Mexico in 2020 relative to the most recent previous year of microdata, netting out seasonal trends in use. Major decreases in health-care use occurred in both countries. Between March and April, 2020, health-care visits per person in Mexico decreased significantly, by an estimated 4·6 (95% UI 2·6–6·5) percentage points, and remained nearly 10 percentage points lower on a monthly basis than 2018 rates until the end of the series. In Peru, health-care visits per person also decreased significantly, with an estimated 48·3 (40·6–56·0) percentage points decrease in April, 2020, relative to March, 2020, and a rebound to more than 20 percentage points lower than March, 2020, from August to November, 2020 (appendix p 25).

**Figure 5:**
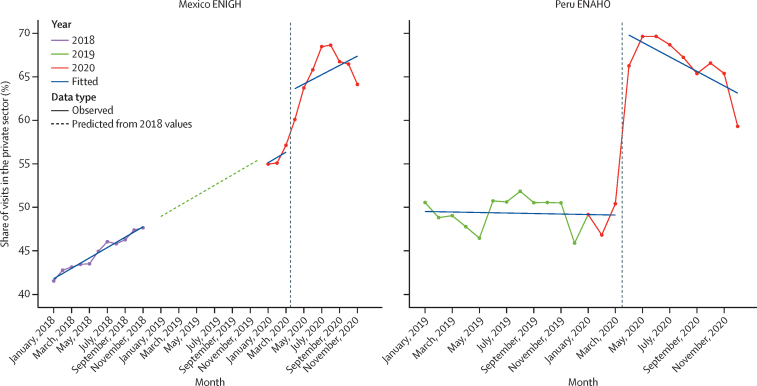
Private sector share of health-care visits in Mexico and Peru, 2018–20 The solid purple, red, and green lines connect the observed datapoints. The blue lines represent the linear fit of the data. The vertical dotted line represents the month that stay-at-home orders were first put in place. ENIGH=Encuesta Nacional de Ingresos y Gastos de los Hogares. ENAHO=La Encuesta Nacional de Hogares.

Changes in the sector of health-care use are depicted in figure 5, which shows that both countries had large increases in the share of health care that occurred in the private sector starting in April, 2020. In Mexico, the private sector share increased by an estimated 7·3 (95% UI 4·3–10·3) percentage points in April, 2020, relative to March, 2020. In Peru, the private sector share increased by an estimated 20·7 (17·3–24·0) percentage points. Full regression results are available in the appendix (pp 31–33).

## Discussion

In 2020, financial hardship due to OOP spending rose in Belarus and Mexico but was unchanged relative to historical trends in Peru, Russia, and Viet Nam. In Mexico and Peru, health-care use decreased in 2020, evidence of increased rates of forgone care. Furthermore, the share of health care that took place in the private sector increased, an indication of differences in the activities of the public versus private sectors in 2020.^52^ In Mexico, OOP spending increased whereas consumption expenditure was steady. In Peru, both OOP spending and consumption expenditure decreased. Insurance coverage decreased substantially in Mexico, which coincided with major changes in the national insurance programme (described below)*.* Insurance coverage was steady in Peru. Overall, the five countries studied had distinct COVID-19 experiences, with COVID-19 deaths ranging from fewer than 75 in Viet Nam to more than 200 000 in Mexico in 2020, that affected financial hardship due to health-care costs.

In Belarus, CHE increased in 2020. Belarus, an upper-middle-income country (GDP per capita of US$6837 in 2019) with total health expenditure (THE) constituting 5·8% of GDP, was the only country in Europe to not adopt stay-at-home orders and require masks in public places,^53^ and had an estimated 19 000 COVID-19 deaths in 2020.^54^, ^55^ It is thus unlikely that forgone care rose in 2020. COVID-19 care probably added to health-care use and OOP spending in Belarus. Although there is very little private health care available,^56^ OOP is the source of nearly a quarter of total health expenditure,^56^, ^57^ and thus additional health-care use could have plausibly resulted in additional health-care spending in Belarus.

In Russia, CHE rates were not higher than predicted by historical trends. Russia, also an upper-middle-income country, but substantially wealthier than Belarus (GDP per capita of $11 536 in 2019) and with similar levels of THE as a share of GDP (5·6%), had the fewest datapoints available for years before 2020, resulting in large uncertainty around the 2020 prediction.^58^ With more than 160 000 COVID-19 deaths in 2020, Russia might have faced health-care capacity constraints and decreases in health-care use for conditions other than COVID-19.^59^ Similar to Belarus, health care takes place predominately in the public sector in Russia. Russia imposed national stay-at-home orders that resulted in decreases in mobility and thus probably produced increases in forgone care, offsetting additional use and spending due to COVID-19.^60^, ^61^

Viet Nam, in contrast to Russia and Belarus, is a lower-middle-income country (GDP per capita of $3491) with lower levels of THE as a share of GDP (4·0%). We similarly observed CHE rates in the range predicted by past trends in Viet Nam. This finding was probably because there was no substantial COVID-19 outbreak in Viet Nam in 2020: fewer than 75 people died of COVID-19 in Viet Nam in 2020. A strict stay-at-home order was imposed for just 3 weeks in April, 2020,^62^ and additional capacity was created to treat patients with COVID-19, making high rates of forgone care unlikely. Viet Nam relies substantially on the private sector for outpatient care (51% of clinics are private), with a more limited role for the private sector in hospital care (15% of hospitals are private).^63^

CHE increased in Mexico in 2020, evidence of worsening financial protection. Mexico is an upper-middle-income country (GDP per capita of $10 145 in 2019) that spends 5·5% of GDP on health. Our analysis suggested that CHE rates rose primarily due to increased OOP health spending and a shift to the private sector, not drops in consumption expenditure. Mexico faced a substantial COVID-19 burden: more than 200 000 people died of COVID-19 in 2020.^64^ We found a substantial shift to health care in the private sector at the time stay-at-home orders were first put in place in March, 2020.^65^ In Mexico, federal policy was to provide COVID-19 hospital care primarily through the public sector.^66^ The estimated shift to the private sector thus potentially reflects both limited capacity in public facilities but also patients’ desire to avoid exposure to COVID-19. Extra OOP spending associated with the private sector was offset somewhat by decreases in use, which have been observed similarly in studies of enrolees in the Mexican Institute of Social Security (IMSS).^67^

Changes in CHE in Mexico cannot be explained by the COVID-19 pandemic alone. By January, 2020, the publicly funded insurance programme for people with relatively low incomes for their country, Seguro Popular*,* which improved access to care and reduced CHE, had been replaced by the National Institute of Health and Wellness programme (and more recently IMSS-Bienestar) in many states.^68^, ^69^ We found that, in 2020, 15·4% (UI –17·9 to –13·0) fewer households reported any insurance coverage as compared with 2018. In 2020, CHE had increased among both insured (to 5·7% [5·1–6·2], a 2·4 percentage point increase) and uninsured (to 5·3% [4·3–6·2], a 2·2 percentage point increase) people since 2018, and at a similar percentage increase (72·1% [45·5–104·7; insured] and 73·4% [23·0–158·1; uninsured]). This finding is consistent with trends ongoing since 2016, which have shown that growth in the insured population has not translated into greater use of health facilities covered by insurance and has been accompanied by increases in OOP spending.^70^ Because of the slightly lower rates of CHE among uninsured people, the increase in the uninsured population might have attenuated the increases in CHE due to the COVID-19 pandemic. Our interrupted time-series analysis provided strong evidence that overall health-care use decreased and the private-sector share of health-care use increased significantly when stay-at-home orders were put in place, not when the change in the insurance programme started in January, 2020. However, we cannot rule out the possibility that the change in the insurance programme created conditions that increased OOP spending and the population's propensity to use the private sector during the COVID-19 pandemic.

Peru's CHE rates were not dissimilar from the rates expected based on past trends, but there is reason to believe that access to health care worsened overall. Peru is a middle-income country, with GDP per capita of $6956 in 2019 (with THE of 4·9%). In Peru, we estimated reductions in health-care visits, OOP spending, and consumption expenditure as the private-sector share of health care increased in 2020. Peru had nearly 100 000 deaths due to COVID-19 in 2020 and one of the highest COVID-19 death rates globally.^71^ Peru had a strict stay-at-home order^72^—mobility decreased more than 90%^73^ and health-care use decreased by 48·3% (UI 40·6–56·0). Steady CHE in Peru thus probably represents a similar amount of financial hardship as expected from past trends, with less health care received overall in 2020 when compared with 2019.

Some limitations pertain to our analysis. First, due to data availability, our analysis was constrained to only five countries and only to 2020, which limits the generalisability of our results to other countries and the years following 2020, which represent distinct periods of the COVID-19 pandemic. We do not have data from countries in the low-income and high-income groups, nor do we have representation from all global regions. Second, our analysis is not causal in nature. We are not able to disentangle the role of COVID-19, including outbreaks, stay-at-home orders, and disruptions to economic activity, from other events in 2020, such as the discontinuation of Seguro Popular in Mexico. Our interrupted time-series analysis uses a short time series; more granular (weekly) data would allow for more robust results. Third, it is also important to consider that collection of data could have been affected by the COVID-19 pandemic. Survey documentation from both surveys reports that no significant differences were found in household characteristics between samples collected during the COVID-19 pandemic and before the pandemic (appendix p 9); however, changes to the data-collection modality, such as telephone interviews in Peru, could have captured different populations than the face-to-face interviews conducted historically. Fourth, our analysis does not capture how financial hardship might have differed across levels of socioeconomic status, as stay-at-home orders and other aspects of the response to the COVID-19 pandemic affected people with more or fewer resources differently. Fifth, the questions on health-care use focused on those who had an illness or injury, and thus health-care use in our study does not capture preventive or well-care visits, limiting our ability to understand how COVID-19 affected patterns of health-care use. The surveys also did not have questions on whether a household member had COVID-19. Sixth, we used linear models for metrics constrained between 0 and 1 (eg, CHE); however, none of our metrics changed substantially enough during the period of interest, such that their uncertainty intervals were close to their natural limits. Finally, we are limited by data availability from considering the role of the denominator of CHE—income or spending—in the countries without microdata. Assessing the denominator is crucial to understanding how financial hardship due to health-care costs changed during the COVID-19 pandemic. In many countries, consumption and income decreased in 2020, and so steady CHE during this time suggests forgone care increased, although more analysis and data are required to affirm this hypothesis. Despite these shortcomings, we believe our analysis represents the best assessment possible of financial protection and its causes in 2020 to date.

Assessing financial hardship due to health-care costs in 2020 is essential to identifying opportunities to make health systems more resilient, particularly in preparation for future pandemics and other shocks.^74^, ^75^ This study provides compelling evidence of worsening financial hardship in Belarus and Mexico in 2020. Moreover, this study suggests that forgone care probably increased in Peru and Russia over the same period. The availability of the private sector in Mexico and Peru might have played a crucial role in mitigating a more substantial decrease in health-care use, underscoring its contribution to the resilience of service coverage during the crisis.^74^, ^75^ However, increases in private-sector use could drive up financial hardship if action to cover private-sector OOP costs is not taken, thus moving countries away from UHC goals. As health systems improve their capacity to respond to future pandemics, they must consider policies and practices that leverage actors across the health system to ensure financial risk protection and service coverage even in times of crisis.

## Data sharing

Estimates of CHE rates in 2020 in Belarus, Mexico, Peru, Russia, and Viet Nam are found in the main manuscript and appendix (pp 17–18). Information about the underlying data sources is available in the appendix (p 8). The code used for this analysis is available on GitHub: https://github.com/ihmeuw/che-covid.

## Declaration of interests

JLD reports support for the present manuscript from the Bill & Melinda Gates Foundation. JLD reports grants or contracts from the Bill & Melinda Gates Foundation, Gates Ventures, and Peterson Center on Healthcare, all outside the submitted work. HJV reports a grant from his university from the Mexican Association of Pharmaceutical Research Industries and direct payment from Roche for attending two meetings of the Roche Mexico Academic Advisory Board regarding long running trends in the Mexican health system; all outside the submitted work. All other authors declare no competing interests.
