# Supplementary material for: Catastrophic health expenditure during the COVID-19 pandemic in five countries: a time-series analysis
Source: Lancet Glob Health. 2023 Sep 19;11(10):e1629–39. doi: 10.1016/S2214-109X(23)00330-3 (PMC10522803; doi:10.1016/S2214-109X(23)00330-3)
Supplement: Supplementary appendix [file mmc1.pdf]

THE LANCET
Global Health

Supplementary appendix

This appendix formed part of the original submission and has been peer reviewed. We post it as supplied by the authors.

Supplement to: Haakenstad A, Bintz C, Knight M, et al. Catastrophic health expenditure during the COVID-19 pandemic in five countries: a time-series analysis. *Lancet Glob Health* 2023; **11:** e1629–39.

Appendix to Catastrophic health expenditure during the COVID-19 pandemic in five countries: a time series analysis

This appendix provides further methodological detail and supplemental figures and tables.

Table of Contents

Author Contributions 3

Preamble 4

List of supplementary tables and figures 5

GATHER Statement 6

Part 1. Estimation of indicators 8

Section 1. Overview 8

Section 1a. History of Catastrophic Health Expenditure 8

Section 1b. Definitions of indicator 8

Section 2. Microdata 8

Section 2a. Sources 8

Section 2b. Processing of input data 9

Section 2c. Data collection in 2020 9

Section 2d. Calculation of CHE 11

Section 3. Drivers of CHE in Mexico and Peru 11

Section 3a. Calculation of any healthcare visit 11

Section 3b. Calculation of any private healthcare visit 11

Section 3c. Calculation of share of healthcare visits in the private sector 11

Section 3d. Calculation of any insurance coverage 12

Section 4. Analysis of trends in CHE and its drivers 12

Section 4a. Model selection 13

Section 4b. Uncertainty 17

Section 4c. CHE Predictions and Observations 17

Section 4d. CHE Driver Predictions 18

Section 5. Interrupted time series analysis of stay-at-home-orders 21

Section 6. Analysis of cross-sectional variation in COVID-19 outcomes 22

Section 6a. COVID-19 and healthcare expenditure and utilization 22

Section 6b. COVID-19 data sources 23

Section 6c. Model specifications 23

Section 6d. Supplementary results 24

Section 6e. Sensitivity analyses 31

Part 2. Online tools 34

Part 3. List of abbreviations 34

References 34

Author Contributions

Managing the estimation process

Annie Haakenstad, Rafael Lozano

Writing the first draft of the manuscript

Annie Haakenstad

Providing data or critical feedback on data sources

Corinne Bintz, Megan Knight

Extracting, cleaning, or cataloging data; designing or coding figures and tables

Corinne Bintz, Horacio Chacon-Torrico, Walter H. Curioso, Joseph L. Dieleman, Annie Haakenstad, Nathaniel J. Henry, Akram Hernández Vásquez, Megan Knight, Judith S. Méndez Méndez, Hector J. Villarreal

Drafting the work or revising it critically for important intellectual content

Annie Haakenstad, Corinne Bintz, Megan Knight, Kelly Bienhoff, Horacio Chacon-Torrico, Walter H. Curioso, Joseph L. Dieleman, Anna Gage, Emmanuela Gakidou, Simon I. Hay, Nathaniel J. Henry, Akram Hernández Vásquez, Judith S. Méndez Méndez, Hector J. Villarreal, Rafael Lozano

Managing the overall research enterprise

Annie Haakenstad, Rafael Lozano

Preamble

This appendix provides methodological detail for estimating catastrophic health expenditure and its drivers as described in the main text but also includes associated supplementary analyses. The appendix is organized into broad sections following the structure of the main paper. This study complies with the Guidelines for Accurate and Transparent Health Estimates Reporting (GATHER) recommendations. It includes detailed indicator modelling write-ups and flowcharts, and information on data sourcing to maximize transparency in our estimation processes and provides a comprehensive account of analytical steps.

List of supplementary tables and figures

Tables

Supplementary table 1. GATHER Checklist
Supplementary table 2. Microdata availability in input data
Supplementary table 3. Sociodemographic and economic characteristics by survey in Mexico ENIGH, 2016-2020
Supplementary table 4a. Normalized RMSE of twelve models predicting CHE (10% and 25%) with out-of-sample predictions of the most recent year of data available prior to 2020
Supplementary table 4b. Average normalized RMSE of twelve models predicting CHE (10% and 25%) using out-of-sample predictions for each year of data available prior to 2020
Supplementary table 5a. CHE (10%) model coefficients
Supplementary table 5b. CHE (25%) model coefficients
Supplementary table 6a. CHE (10%) 2020 predictions and observations
Supplementary table 6b. CHE (25%) 2020 predictions and observations
Supplementary table 7a. Average consumption (PPP 2021) model coefficients
Supplementary table 7b. OOP health spending (PPP 2021) model coefficients
Supplementary table 7c. Share of individuals with any insurance model coefficients
Supplementary table 8a. Average consumption (PPP 2021) 2020 predictions and observations
Supplementary table 8b. OOP health spending 2021 purchasing power parity (PPP 2021) 2020 predictions and observations
Supplementary table 8c. Share of individuals with any insurance 2020 predictions and observations
Supplementary table 9a: Interrupted time series regressions, Mexico
Supplementary table 9b: Interrupted time series regressions, Peru
Supplementary table 10. Linear probability model of healthcare expenditure and utilization on COVID-19 deaths
Supplementary table 11. Binomial model of healthcare expenditure and utilization on COVID-19 deaths
Supplementary table 12. Linear probability model of healthcare expenditure and utilization on COVID-19 hospitalizations
Supplementary table 13. Binomial model of healthcare expenditure and utilization on COVID-19 hospitalizations
Supplementary table 14. Linear probability model of healthcare expenditure and utilization on COVID-19 cases
Supplementary table 15. Binomial model of healthcare expenditure and utilization on COVID-19 cases
Supplementary table 16. Linear probability model of healthcare expenditure and utilization on COVID-19 tests
Supplementary table 17. Binomial model of healthcare expenditure and utilization on COVID-19 tests

Figures

Supplementary figure 1. Socioeconomic stratum by type of questionnaire, phone or in person, in Peru ENAHO 2020
Supplementary figure 2. Share of visits at a pharmacy
Supplementary figure 3. Share of visits in the private sector excluding pharmacies
Supplementary figure 4. Predicted and observed values of CHE (10% and 25%) using Ensemble 2 (models 2-5)
Supplementary figure 5. Predicted and observed values of CHE (10% and 25%) using Ensemble 3 (models 2 and 4)
Supplementary figure 6. Predicted and observed values of CHE (10% and 25%) using Ensemble 4 (models 4 and 5)
Supplementary figure 7. Predicted and observed values of CHE (10% and 25%) using Ensemble 5 (models 1-6)
Supplementary figure 8. Predicted and observed values of CHE utilization drivers, Ensemble 2 (models 2-5 for Peru)
Supplementary figure 9. Association between CHE and CHE drivers with subnational variation in the log COVID-19 death rate in Mexico and Peru, 2020
Supplementary figure 10. Coefficients from sensitivity analysis of COVID-19 mortality regressions using different cut-off thresholds
Supplementary figure 11. P-values from sensitivity analysis of COVID-19 mortality regressions using different cut-off thresholds

GATHER Statement

This study complies with the Guidelines for Accurate and Transparent Health Estimates Reporting (GATHER) recommendations.[1] We have documented the steps involved in our analytical procedures and detailed the data sources used in compliance with the GATHER. For additional GATHER reporting, please refer to Supplementary table 1 on pages 6-8.

Supplementary table 1. GATHER Checklist

#	GATHER checklist item	Description of compliance	Reference
**Objectives and funding**			
1	Define the indicator(s), populations (including age, sex, and geographic entities), and time period(s) for which estimates were made.	Description of indicators, definitions, relevant time periods, and populations in paper and appendix.	Main Text Methods; Main Text, Table 1; Appendix, Part 1, Section 1.
2	List the funding sources for the work.	Funding sources listed in paper.	Main Text Summary.
**Data inputs**			
For all data inputs from multiple sources that are synthesized as part of the study:			
3	Describe how the data were identified and how the data were accessed.	Narrative description of data seeking methodology provided.	Appendix, Part 1, Section 2.
4	Specify the inclusion and exclusion criteria. Identify all ad-hoc exclusions.	Narrative about inclusion and exclusion criteria by data type provided in linked materials.	Main Text Methods; Appendix, Part 1, Section 2.
5	Provide information on all included data sources and their main characteristics. For each data source used, report reference information or contact name/institution, population represented, data collection method, year(s) of data collection, sex and age range, diagnostic criteria or measurement method, and sample size, as relevant.	Description of metadata for data sources and links to their sources for public download.	Main Text Methods; Appendix, Part 1, Section 2; Appendix Part 1, Section 2
6	Identify and describe any categories of input data that have potentially important biases (eg, based on characteristics listed in item 5).	Summary of known biases included in paper and appendix.	Main Text Methods; Main Text Limitations; Appendix, Part 1, Section 3.
For data inputs that contribute to the analysis but were not synthesized as part of the study:			
7	Describe and give sources for any other data inputs.	We describe in the text our additional data sources as well as provide links where the data can be downloaded.	Main Text Methods; Appendix, Part 1, Section 2; Appendix Part 1, Section 2.
For all data inputs:			
8	Provide all data inputs in a file format from which data can be efficiently extracted (eg, a spreadsheet as opposed to a PDF), including all relevant meta-data listed in item 5. For any data inputs that cannot be shared due to ethical or legal reasons, such as third-party ownership, provide a contact name or the name of the institution that retains the right to the data.	We describe in the text our additional data sources as well as provide links where the data can be downloaded.	Main Text Methods; Appendix Part 1, Section 2.
**Data analysis**			
9	Provide a conceptual overview of the data analysis method. A diagram may be helpful.	We describe step-by-step our process in the methods section of the main text with more details in the appendix.	Main Text Methods; Appendix, Part 1, Section 3.
10	Provide a detailed description of all steps of the analysis, including mathematical formulae. This description should cover, as relevant, data cleaning, data pre-processing, data adjustments and weighting of data sources, and mathematical or statistical model(s).	We describe step-by-step our process in the methods section of the main text with more details in the appendix.	Main Text Methods; Appendix, Part 1, Sections 2 and 3.
11	Describe how candidate models were evaluated and how the final model(s) were selected.	We describe step-by-step our process in the methods section of the main text with more details in the appendix.	Main Text Methods; Appendix, Part 1 Sections 4, 5, and 6.
12	Provide the results of an evaluation of model performance, if done, as well as the results of any relevant sensitivity analysis.	We describe step-by-step our process in the methods section of the main text with more details in the appendix.	Main Text Methods; Appendix, Part 1 Section 6.
13	Describe methods for calculating uncertainty of the estimates. State which sources of uncertainty were, and were not, accounted for in the uncertainty analysis.	We describe step-by-step our process in the methods section of the main text with more details in the appendix.	Main Text Methods; Appendix, Part 1 Sections 4, 5, and 6.
14	State how analytic or statistical source code used to generate estimates can be accessed.	Access statement provided.	Main Text, Data Sharing
**Results and Discussion**			
15	Provide published estimates in a file format from which data can be efficiently extracted.	Tables available in the supplementary appendix.	Appendix, Part 1 Sections 4, 5, and 6

**16**	Report a quantitative measure of the uncertainty of the estimates (e.g., uncertainty intervals).	Uncertainty intervals are provided with all results.	Main text results, Main text tables 1 and 2.
**17**	Interpret results in light of existing evidence. If updating a previous set of estimates, describe the reasons for changes in estimates.		Main Text, Discussion.
**18**	Discuss limitations of the estimates. Include a discussion of any modelling assumptions or data limitations that affect interpretation of the estimates.	Discussion of limitations provided in the main text.	Main Text, Methods and Discussion.

Part 1. Estimation of indicators

Section 1. Overview

Section 1a. History of Catastrophic Health Expenditure

The World Bank and the World Health Organization have been reporting the incidence of catastrophic health expenditure (CHE) at the global level since 2015 when the United Nations launched the Sustainable Development Goals (SDGs). SDG Indicator 3.8.2 is formally defined as the proportion of a population with large household expenditures on health as a share of total income or household consumption expenditure, commonly referred to as CHE.[2]

> SDG Goal 3: Ensure healthy lives and promote well-being for all at all ages.
>
> Target 3.8: Achieve universal health coverage, including financial risk protection, access to quality essential health-care services and access to safe, effective, quality and affordable essential medicines and vaccines for all.
>
> SDG Indicator 3.8.2: Proportion of population with large household expenditures on health as a share of total household expenditure or income

Section 1b. Definitions of indicator

Catastrophic health expenditure (CHE) is a common measure of financial risk protection that occurs when out-of-pocket (OOP) health expenditure exceeds a pre-defined share of household income or household consumption spending. In this case, the thresholds for CHE are 10 and 25 percent of total household expenditure or income to align with SDG Indicator 3.8.2.

Household equation:
CHE = 1 if

$$\frac{\text{Health expenditure}}{\text{Total household consumption expenditure}} > 0.1 \text{ or } 0.25$$

Population equation:

$$\text{CHE rate} = \frac{\sum_{i=1}^{N} \text{CHE}}{\text{N}}$$

Section 2. Microdata

Section 2a. Sources

The present study used two primary types of input data:

(1) individual and household level survey microdata; and
(2) tabulated data from the Health Equity and Financial Protection Indicator (HEFPI) database.

Microdata for 2020 was publicly available for two countries: Mexico and Peru. In Mexico, the National Survey of Household Income and Expenditure (ENIGH) has been implemented between August and November biennially since 1984.[3] In Peru, the National Household Survey on Living Conditions and Poverty (ENAHO) has been conducted year-round since 1995.[4]

Tabulated data for 2020 describing CHE were used for Vietnam, Russia, and Belarus. These estimates were derived from long-running surveys in the respective countries. Since 1995, the Belarus Household Survey has been conducted annually and over the course of the year.[5] The Russia Household Budget Survey has been conducted at the household level quarterly and in a continuous cycle by the Federal State Statistics Service since 1952.[6] Data

from Vietnam originated from the Living Standards Measurement Study, which has collected household expenditure data since 1992.[7] For Belarus, we used the CHE estimates reported by the National Statistical Committee of the Republic of Belarus. In Russia and Vietnam, we used CHE estimates reported by the Global Monitoring Report on Financial Protection in Health published by the World Bank and World Health Organization (WHO) in 2021 and made available in the HEFPI database.[8]

Supplementary table 2. Microdata availability in input data

Country	Source	Microdata
Mexico	ENIGH	Available
Peru	ENAHO	Available
Vietnam	HEFPI	Not available
Russia	HEFPI	Not available
Belarus	HEFPI	Not available

Section 2b. Processing of input data

Microdata from Peru's ENAHO and Mexico's ENIGH surveys were available at the household and individual level. Health expenditure was calculated by combining subcategories of health spending available at the household level, and total consumption expenditure was calculated by combining subcategories of consumption expenditure. The individual level data provided data on education level, age, health insurance coverage, sex, and healthcare visits. The household level file provided data on health spending, rural or urban location, and consumption expenditure. In these surveys, different recall periods were used to ask about spending for different categories. When the recall periods were less than annual (a one-year recall period), we multiplied the result by a scalar to annualize results (e.g., 12 if the recall period was one month). Health spending from the household level file from Mexico and Peru were available in subcategories, which we summed to calculate total out-of-pocket (OOP) health expenditure. Household and individual level files were merged on household identifying variables. Survey weights were applied to calculate representative summary measures of the population, as seen in the manuscript's table 1.

Section 2c. Data collection in 2020

Across the world, the COVID-19 pandemic, and responses thereto, disrupted face-to-face data collection. Here we describe how that affected the data we used in our analysis.

Peru. In Peru, the ENAHO is typically conducted all year round and this continued despite the pandemic. In 2020, surveys were conducted face-to-face between January and mid-March, and October and December.[9] Between late March and September, however, phone interviews and other alternative methods were used to collect data. The National Institute of Statistics and Informatics in Peru made extensive effort, including many different follow-up methods, to ensure response rates in the phone interviews were similar to the face-to-face surveys. Survey documentation from the Peru ENAHO in 2020 reports that no significant differences were found in household characteristics between those surveyed via phone and in person except for the type of fuel used for cooking. However, significant differences were found in sex and age group between the population surveyed via phone versus in person. We did not find substantial differences in the percent of households in each socioeconomic stratum by type of questionnaire, shown below in Supplementary figure 1.

Supplementary figure 1. Socioeconomic stratum by type of questionnaire, phone or in person, in Peru ENAHO 2020

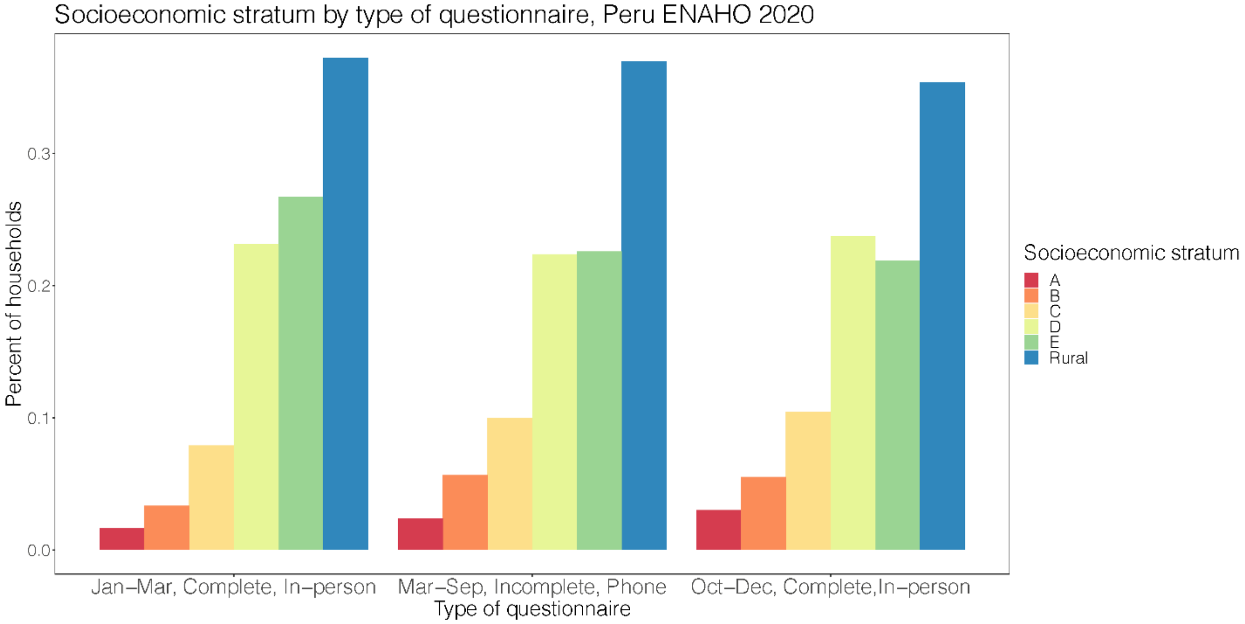

Mexico. In Mexico, data collection in 2020 proceeded similarly to prior years, with face-to-face interviews comprising the main data collection modality.[10] In Mexico, data collection for the ENIGH has historically occurred in the last quarter of the year, August through November. Data collection for ENIGH in 2020 remained face-to-face and occurred between August 8th and November 28th, 2020. Precautionary measures were taken to increase the sample size by 20% in anticipation of higher rates of non-response. At the national level, the non-response amounted to 16.7%. Supplementary table 3 shows that the ENIGH 2020 sample is comparable to previous samples observed in 2016 and 2018.

Supplementary table 3. Sociodemographic and economic characteristics by survey in Mexico ENIGH, 2016-2020

**Total households and members (absolute)**	**ENIGH 2016**	**ENIGH 2018**	**ENIGH 2020**	**Percentage change 2016-2018**	**Percentage change 2018-2020**
total households	32 974 661	34 400 515	35 749 659	4.3*	3.9*
Total household members1	120 801 511	123 836 081	126 760 856	2.5*	2.4*
**Characteristic sociodemographic and economic (average people)**	**ENIGH 2016**	**ENIGH 2018**	**ENIGH 2020**	**Percentage change 2016-2018**	**Percentage change 2018-2020**
Household size1	3.66	3.60	3.55	-1.7*	-1.5*
Household members under 15 years of age1	1.00	0.93	0.85	-6.7*	-9.1*
Household members from 15 to 64 years old1	2.38	2.37	2.37	-0.4	-0.1
Household members aged 65 and over1	0.29	0.30	0.33	4.6*	10.9*
Economically active household members aged 15 and over	1.74	1.75	1.73	0.4	-1.0*
Household members aged 15 and over who are not economically active	0.93	0.92	0.97	-0.4	5.1*
Earners per household	2.45	2.38	2.25	-2.9*	-5.6*
Busy household members	1.69	1.70	1.64	0.5	-3.3*

Source: Mexico ENIGH survey.[10]

Belarus, Russia and Vietnam. Detailed documentation on 2020 data collection procedures for the other countries was not available. However, in Belarus, as we note in the discussion in the main text, little-to-no social distancing occurred, which suggests that there is little reason to believe that response rates differed as compared to prior years due to the pandemic. Similarly, Vietnam had minimal disruptions to in-person interaction because COVID-19 cases remained low and so we do not have reason to believe response rates differed in this case as well. We were unable to identify documentation describing the data collection procedures employed in Russia in 2020 and how they may have differed from data collected prior to 2020.

Section 2d. Calculation of CHE

To calculate CHE, we computed the share of consumption expenditure attributed to OOP health expenditure. We then created a binary variable for the CHE thresholds of 10% and 25%, assigning a household 1 if their health expenditure was greater than 10% or 25% of their total consumption expenditure and assigned a 0 otherwise. Using the binary values assigned and survey weights, we calculated the percentage of households with CHE using each threshold.

Section 3. Drivers of CHE in Mexico and Peru

Section 3a. Calculation of any healthcare visit

In Mexico, healthcare visits were among individuals who reported pain, discomfort, illness or an accident that prevented them from performing daily activities in the past year that received care anywhere (MOH health centers, MOH hospital, IMSS, IMSS-Prosperpa/IMSS-Bienestar, ISSSTE, ISSTE-Estatal, PEMEX, private clinics and hospitals, pharmacy, INSABI). In Peru, healthcare visits were among individuals who reported symptoms of cough, headache, fever, nausea, other illness such as flu or colitis, relapse of chronic illness, an accident, or symptoms of COVID-19 and received care anywhere (MINSA health center, post, or hospital, CLAS health centers or posts, ESSALUD health center, ESSALUD hospital, FF.AA. and/or National police hospital, private medical doctor's office, private clinic, or pharmacy).

Section 3b. Calculation of any private healthcare visit

In Mexico, individuals who reported pain, discomfort, illness, or an accident that prevented them from performing daily activities in the past year that received care in a private healthcare setting (private clinics and hospitals, pharmacy). In Peru, individuals who reported symptoms of cough, headache, fever, nausea, other illness such as flu or colitis, relapse of chronic illness, an accident, or symptoms of COVID-19 and received care at a private setting (private doctor's office, private clinic, pharmacy).

Section 3c. Calculation of share of healthcare visits in the private sector

In both Mexico and Peru, the proportion of individuals who had a visit in the private sector was among all individuals who had a healthcare visit by month. In Peru, the month is ascertained from the date of the interview, since the recall period for healthcare visits was the last four weeks. In Mexico, the month is ascertained from the reported date of the healthcare visit. We also calculated the proportion of individuals who had a visit in the private sector excluding pharmacies of all individuals who had a healthcare visit by month, and the proportion of individuals who had a visit at a pharmacy of all individuals who had a healthcare visit by month. We note that there are substantially different patterns in Mexico and Peru when considering the role of private pharmacies separately from other types of private provider, as shown in the figures below.

Supplementary figure 2. Share of visits at a pharmacy

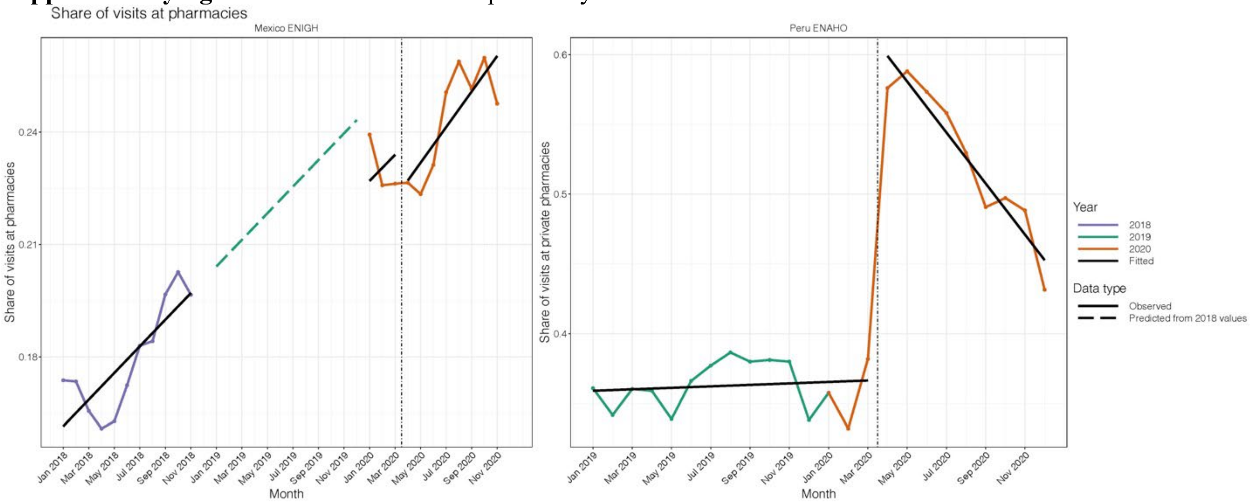

Supplementary figure 3. Share of visits in the private sector excluding pharmacies

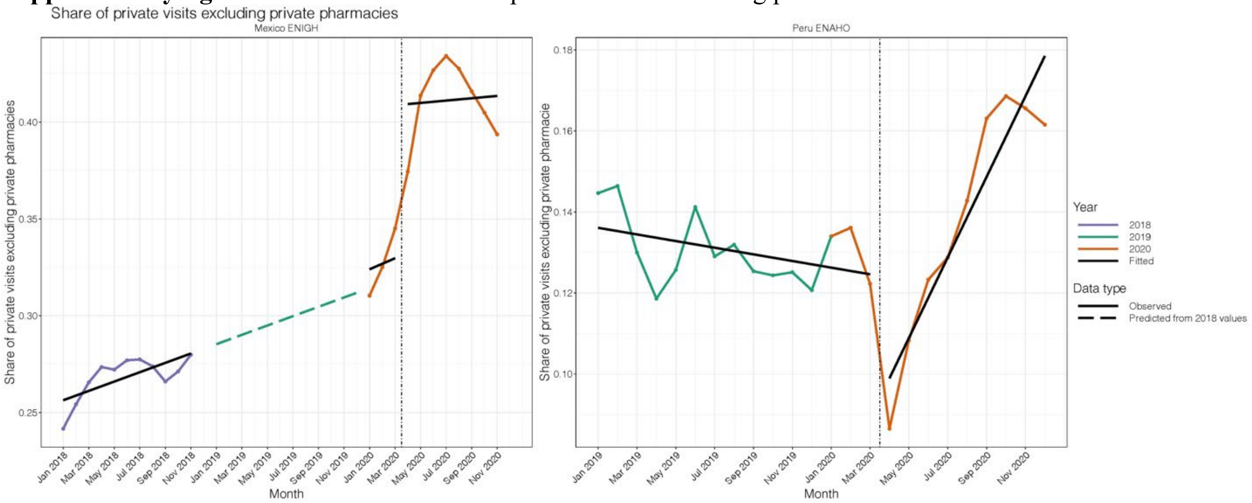

Section 3d. Calculation of any insurance coverage

To construct the insurance coverage metric, we leveraged individual data measuring public and private insurance coverage in Mexico. In Mexico, a household was considered to have insurance coverage if any individual in the household reported having public or private insurance coverage (INSABI, IMSS, ISSSTE, ISSSTE-Estatal, PEMEX, IMSS-Prospera/IMSS-Bienestar), otherwise a household was designated as having no insurance coverage. Similarly, we leveraged individual level data measuring public and private insurance coverage in Peru. In Peru, a household was considered to have insurance coverage if any individual in the household reported having public or private insurance coverage (ESSALUD, private health insurance, insurance from a healthcare provider, FF.AA/Police, Seguro Integral de Salud (SIS), university, private school), otherwise a household was designated as having no insurance coverage. By applying survey weights, we calculated the percentage of households with any insurance coverage.

<u>Section 4. Analysis of trends in CHE and its drivers</u>

We employed a stratified ensemble model to make predictions of CHE in 2020 for each of the five countries based on previous trends. For each of the five countries, we ran the following ordinary least squares regressions of CHE on the observed available data from 2006 to 2019 as shown in equations (1) and (2).
Level regressed on lag-level, first-order autoregressive model:

$$\Upsilon_t = \beta_0 + \beta_1 \Upsilon_{t-1} + \varepsilon_t \quad (1)$$

Difference regressed on lag-difference, differenced first-order model:

$$Diff_{\Upsilon_t} = \beta_0 + \beta_1 Diff_{\Upsilon_{t-1}} + \varepsilon_t \quad (2)$$

Where Υ_t is the CHE value and $Diff_{\Upsilon_t}$ is the differenced value of the CHE value. Both models were run with and without a constant, β_0. For each model, we extracted 1000 coefficients from the variance-covariance matrix. We combined the 1000 predictions from each model for a total of 4000 predictions and took the 2.5th and 97.5th percentiles as well as the mean to construct a point prediction and 95% confidence interval of CHE in 2020. We compared the observed value and the predicted value to note any deviations in CHE.

Section 4a. Model selection

We undertook a model selection process to determine our prediction approach. In addition to the models in equations (1) and (2) with and without an intercept term, we tested a version of the model with a linear term for time (Models 1 and 6) for both the level and differenced CHE. We tested a differenced model with two-lags (Model 7). We also tested the (ultimately selected) ensemble model based on using models 2-5. We based on our selection on the normalized out-of-sample root mean square error (RMSE) for the most recent data point prior to 2020 (2019 in Peru and Belarus, 2018 in Mexico and Vietnam and 2014 in Russia). We ran each of the models described below without 2020 and the other most recent prior point, predicted the value of the most recent prior point and compared it to the observed value. The normalized CHE is calculated by dividing the root squared difference between the prediction and observed by the observed CHE value. The normalized RMSE allows us to more easily compare CHE 10% and CHE 25% and more easily compare across countries with substantially different levels of CHE. Across the normalized RMSE results, the ensemble models performs better OOS for CHE (10%) than any individual model. Ultimately, we selected ensemble 2 because it draws from all models with lags and demonstrates good performance in the countries with the highest and lowest data coverage. We note that ensemble 4 out-perform ensemble 2 in terms of normalized OOS RMSE, but the large (non-significant) increase in CHE predicted at the mean in Russia shows that this model does not perform well in both high- and low-data coverage countries. Ensembles 3 and 5 also outperform ensemble 2 for other measures of OOS RMSE in tables 4a and 4b. For this reason, we depict the results of ensembles 2-5 below, which show that the overall findings of a significant change in CHE in Mexico (10% and 25%) and Belarus (10%) and no other countries are robust to the selection of ensembles.

Supplementary table 4a. Normalized RMSE of twelve models predicting CHE (10% and 25%) with out-of-sample predictions of the most recent year of data available prior to 2020

	Model 1: level CHE ~ year	Model 2: Level CHE ~ lag CHE, with intercept	Model 3: Diff CHE ~ Lag Diff CHE, with intercept	Model 4: Level CHE ~ lag CHE, without intercept	Model 5: Diff CHE ~ Lag Diff CHE, without intercept	Model 6: Diff CHE ~ year	Model 7: Diff CHE ~ Lag Diff CHE + Double Lag Diff CHE	Ensemble 1: Model 2 and Model 3	Ensemble 2: Models 2-5	Ensemble 3: Models 2 & 4	Ensemble 4: Models 4 & 5	Ensemble 5: Models 1-6, weighted by RMSE
CHE 10%	0.2118	0.0948	0.095	0.1411	0.0934	0.1107	0.1114	0.0908	0.0894	0.0943	0.088	0.0983
CHE 25%	0.286	0.2192	0.2052	0.197	0.1506	0.1815	0.2426	0.1932	0.1782	0.1974	0.1631	0.1645
Both	0.2489	0.157	0.1501	0.169	0.122	0.1461	0.177	0.142	0.1338	0.1459	0.1256	0.1314

Supplementary table 4b. Average normalized RMSE of twelve models predicting CHE (10% and 25%) using out-of-sample predictions for each year of data available prior to 2020

	Model 1: level CHE ~ year	Model 2: Level CHE ~ lag CHE, with intercept	Model 3: Diff CHE ~ Lag Diff CHE, with intercept	Model 4: Level CHE ~ lag CHE, without intercept	Model 5: Diff CHE ~ Lag Diff CHE, without intercept	Model 6: Diff CHE ~ year	Model 7: Diff CHE ~ Lag Diff CHE + Double Lag Diff CHE	Ensemble 1: Model 2 and Model 3	Ensemble 2: Models 2-5	Ensemble 3: Models 2 & 4	Ensemble 4: Models 4 & 5	Ensemble 5: Models 1-6, weighted by RMSE
CHE 10%	0.1502	0.1417	0.1461	0.1613	0.1422	0.1572	0.1654	0.1434	0.1372	0.1365	0.1443	0.1297
CHE 25%	0.2771	0.3458	0.3693	0.2935	0.2877	0.3565	0.3676	0.3435	0.3061	0.3084	0.2735	0.3061
Both	0.2137	0.2438	0.2577	0.2274	0.215	0.2568	0.2665	0.2435	0.2217	0.2225	0.2089	0.2077

Supplementary table 5a. CHE (10%) model coefficients

		Level CHE ~ year (Model 1)	Lag model with constant (model 2)	Difference model with constant (model 3)	Lag model without constant (model 4)	Difference model without constant (model 5)	Diff CHE ~ year (Model 6)
Belarus	Constant	-91884	37.3	55.5	-	-	-4128
	Coefficient	46.0	0.993	-0.116	1.05	0.203	2.07
	P value (coefficient)	<.001	<.001	0.679	<.001	0.458	0.694
	N	16	16	14	16	14	15
Mexico	Constant	75256	215	3.68	-	-	-21098
	Coefficient	-37.2	0.298	0.374	0.821	0.334	10.5
	P value (coefficient)	0.018	0.011	0.047	<.001	0.018	0.025
	N	7	6	5	6	5	6
Peru	Constant	-28646	391	6.9	-	-	17712
	Coefficient	14.7	0.607	0.204	1	0.227	-8.8
	P value (coefficient)	0.065	0.016	0.543	<.001	0.465	0.0679
	N	14	13	12	13	12	13
Russia	Constant	-41383	200	32.6	-	-	-34779
	Coefficient	20.8	0.545	-0.865	1.2	-0.815	17.3
	P value (coefficient)	0.010	0.125	0.375	<.001	0.318	0.634
	N	6	6	4	6	4	5
Viet Nam	Constant	121640	276	-38.5	-	-	-16548
	Coefficient	-59.9	0.746	-0.0370	0.964	0.240	8.2
	P value (coefficient)	<.001	0.043	0.949	<.001	0.584	0.286
	N	8	8	6	8	6	7

Supplementary table 5b. CHE (25%) model coefficients

		Level CHE ~ year (model 1)	Lag model with constant (model 2)	Difference model with constant (model 3)	Lag model without constant (model 4)	Difference model without constant (model 5)	Diff CHE ~ year (model 6)
Belarus	Constant	-7093	6.70	5.30	-	-	-140
	Coefficient	3.54	0.865	-0.606	1.02	-0.5	0.071
	P value (coefficient)	<.001	<.001	0.0232	<.001	0.065	0.923
	N	16	16	14	16	14	15
Mexico	Constant	18956	53.6	1.1	-	-	-6494
	Coefficient	-9.39	0.181	0.175	0.771	0.142	3.22
	P value (coefficient)	0.037	0.308	0.570	0.001	0.54	0.047
	N	7	6	5	6	5	6
Peru	Constant	-8928	73.7	3.61	-	-	3101
	Coefficient	4.51	0.476	0.00241	1.01	0.055	-1.54
	P value (coefficient)	0.003	0.050	0.994	<.001	0.863	0.195
	N	14	13	12	13	12	13
Russia	Constant	-8416	33.5	-0.049	-	-	-7435
	Coefficient	4.21	0.205	-0.835	1.05	-0.835	3.70
	P value (coefficient)	0.049	0.707	0.378	0.030	0.261	0.747
	N	6	6	4	6	4	5
Viet Nam	Constant	39512	83.8	-12.4	-	-	-6968
	Coefficient	-19.5	0.680	-0.039	0.953	0.195	3.46
	P value (coefficient)	0.002	0.067	0.951	<.001	0.690	0.257
	N	8	8	6	8	6	7

Supplementary figure 4. Predicted and observed values of CHE (10% and 25%) using Ensemble 2 (models 2-5)

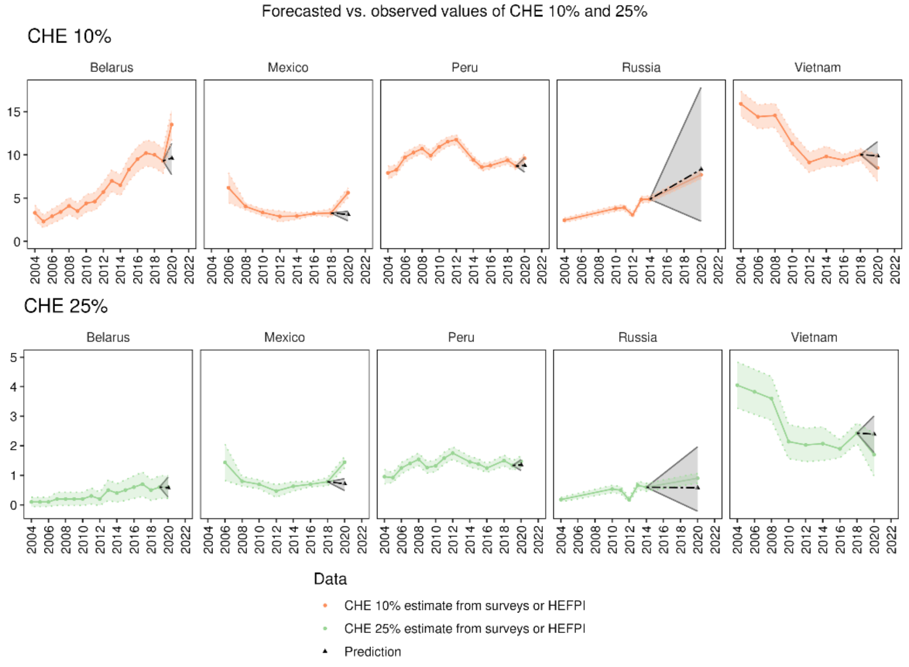

Supplementary figure 5. Predicted and observed values of CHE (10% and 25%) using Ensemble 3 (models 2 and 4)

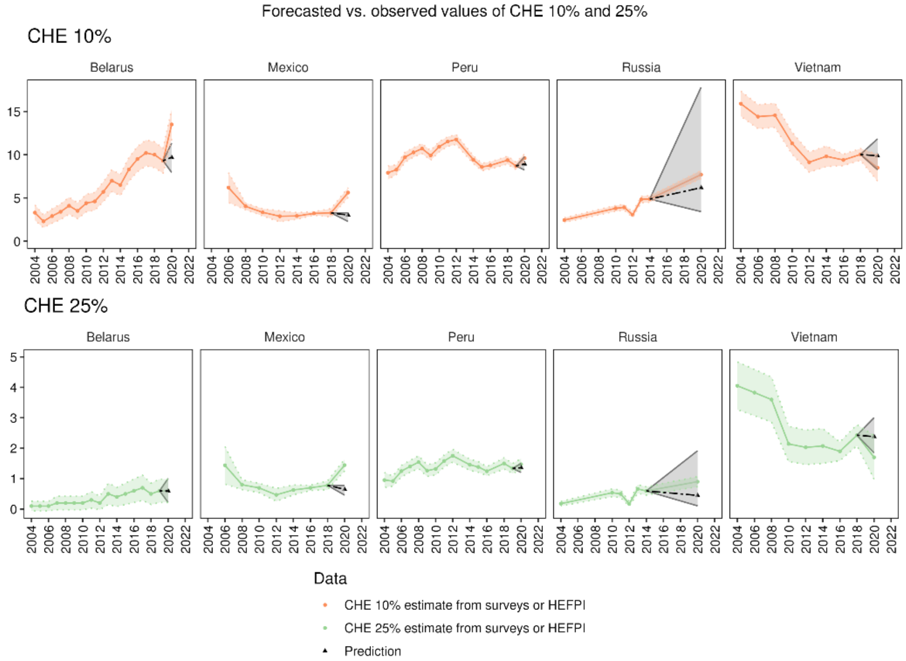

Supplementary figure 6. Predicted and observed values of CHE (10% and 25%) using Ensemble 4 (models 4 and 5)

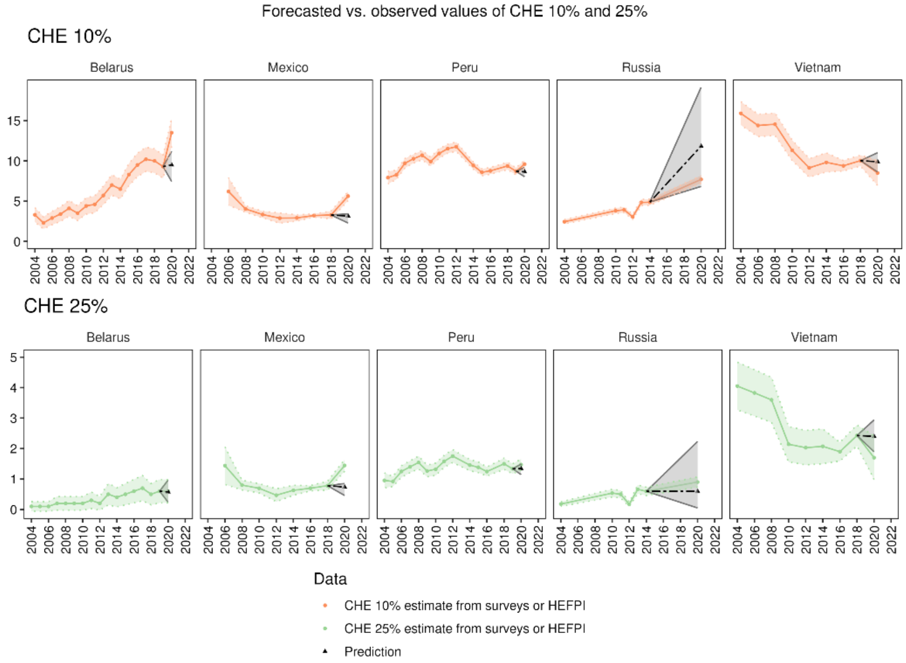

Supplementary figure 7. Predicted and observed values of CHE (10% and 25%) using Ensemble 5 (models 1-6)

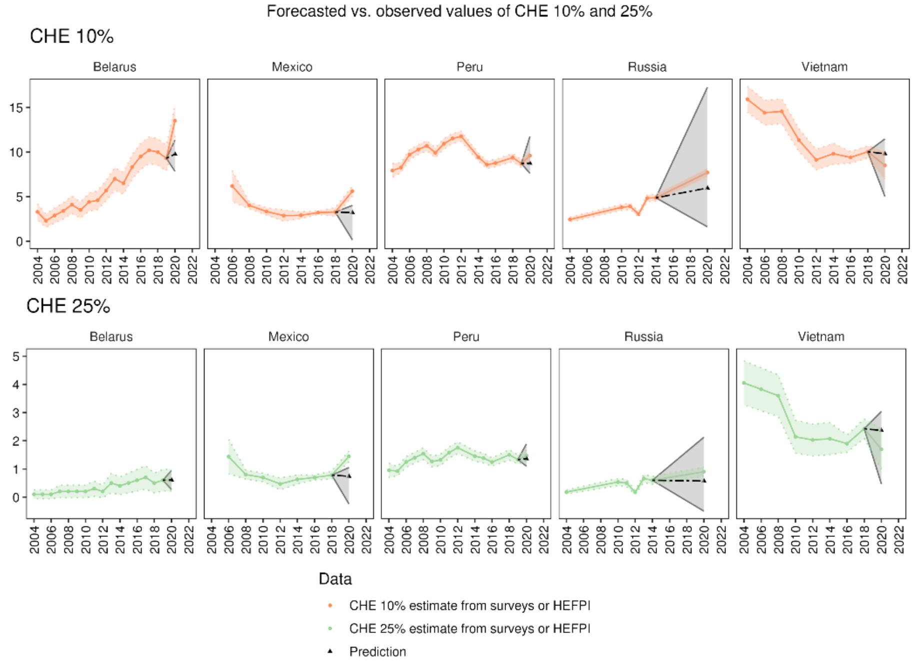

Section 4b. Uncertainty

We quantify the data uncertainty in the observed values and quantify both data and model uncertainty in our predictions. We simulate data uncertainty based on the standard error of the sample estimate of CHE by country and year. Although we did not have the microdata for Belarus, Russia, and Vietnam, we are able to impute the standard error with the reported CHE value and the sample size N. Re-arranging the terms of the basic standard error equation, we use the following to calculate the standard errors in the countries where no microdata are available:

$$SE = \sqrt{\frac{(N*CHE*(1-CHE))}{N}} * 1.96$$

Where CHE is the mean proportion of CHE 10% and 25% as reported in the tabulated data. We simulate 1000 draws of CHE and take the 2.5 and 97.5 percentiles to quantify the uncertainty in the observed data.

Section 4c. CHE Predictions and Observations

In Tables 6a and 6b below, we present the results underpinning Figure 1 in the main text, depicting both the observed 2020 value for CHE as well as the CHE value predicted based on our model.

Supplementary table 6a. CHE (10%) 2020 predictions and observations

Country	2020 Prediction	2020 Observed
Belarus	9.7<br>(7.7 to 11.3)	13.5<br>(11.8 to 15.2)
Mexico	3.2<br>(2.4 to 3.5)	5.6<br>(5.1 to 6.2)
Peru	8.7<br>(8.0 to 9.7)	9.6<br>(9.1 to 10.1)
Russia	7.0<br>(2.3 to 17.8)	7.7<br>(7.2 to 8.2)
Vietnam	9.9<br>(8.3 to 11.5)	8.5<br>(7.0 to 10.0)

Supplementary table 6b. CHE (25%) 2020 predictions and observations

Country	2020 Prediction	2020 Observed
Belarus	0.6<br>(0.3 to 0.9)	0.6<br>(0.2 to 1.0)
Mexico	0.7<br>(0.5 to 0.9)	1.4<br>(1.3 to 1.6)
Peru	1.4<br>(1.2 to 1.5)	1.5<br>(1.3 to 1.7)
Russia	0.5<br>(-0.2 to 2.0)	0.9<br>(0.7 to 1.1)
Vietnam	2.4<br>(1.8 to 3.0)	1.7<br>(1.0 to 2.4)

Section 4d. CHE Driver Predictions

We employed the same two models and ensemble approach (Ensemble 2) to compare the predicted value for drivers of CHE in Mexico and Peru with the observed value, including average consumption, out of pocket health spending, and share of individuals with any insurance.

Supplementary table 7a. Average consumption (PPP 2021) model coefficients

Country		Lag model with constant (model 2)	Difference model with constant (model 3)	Lag model without constant (model 4)	Difference model without constant (model 5)
Mexico	Constant	13135	-1188	-	-
	Coefficient	0.329	-0.478	0.95436	0.1417
	P value (coefficient)	0.024	0.256	<0.001	0.237
	N	6	6	6	5
Peru	Constant	2394	229	-	-
	Coefficient	0.866	0.009	1.009	0.131
	P value (coefficient)	<0.001	0.975	<0.001	0.649
	N	13	12	13	12

Supplementary table 7b. OOP health spending (PPP 2021) model coefficients

Country		Lag model with constant (model 2)	Difference model with constant (model 3)	Lag model without constant (model 4)	Difference model without constant (model 5)
Mexico	Constant	271.6	-71.8	-	-
	Coefficient	0.291	-0.213	0.82571	0.26886
	P value (coefficient)	0.00195	0.654	<0.001	0.0199
	N	6	6	6	5
Peru	Constant	216.0	10.6	-	-
	Coefficient	0.720	0.231	1.0107	0.2847
	P value (coefficient)	<.001	0.471	<0.001	0.346
	N	13	12	13	12

Supplementary table 7c. Share of individuals with any insurance model coefficients

Country		Lag model with constant (model 2)	Difference model with constant (model 3	Lag model without constant (model 4)	Difference model without constant (model 5)
Mexico	Constant	0.334	-0.025	-	-
	Coefficient	0.61	1.02	1.055	0.661
	P value (coefficient)	0.003	0.082	<0.001	0.0189
	N	5	4	5	4
Peru	Constant	0.070	0.027	-	-
	Coefficient	0.934	0.231	1.047	0.579
	P value (coefficient)	<.001	0.451	<0.001	0.0383
	N	13	12	13	12

Supplementary table 8a. Average consumption (PPP 2021) 2020 predictions and observations

Country	2020 Prediction	2020 Observed
Mexico	19,067<br>(16,730 to 21,199)	17,670<br>(16,117 to 19,223)
Peru	17,763<br>(17,293 to 18,263)	14,370<br>(14,100 to 14,640)

Supplementary table 8b. OOP health spending 2021 purchasing power parity (PPP 2021) 2020 predictions and observations

Country	2020 Prediction	2020 Observed
Mexico	370<br>(277 to 444)	552<br>(497 to 608)
Peru	733.82<br>(679 to 780)	628<br>(601 to 655)

Supplementary table 8c. Share of households with any insurance coverage among household members 2020 predictions and observations

Country	2020 Prediction	2020 Observed
Mexico	0.83<br>(0.76 to 0.90)	0.69<br>(0.68 to 0.71)
Peru	0.80<br>(0.77 to 0.84)	0.77<br>(0.77 to 0.78)

We also produced a figure of the drivers of CHE and CHE using just the difference model without a constant (model 5).

Supplementary figure 8: Predicted and observed values of CHE utilization drivers, Ensemble 2 (models 2-5 for Peru)

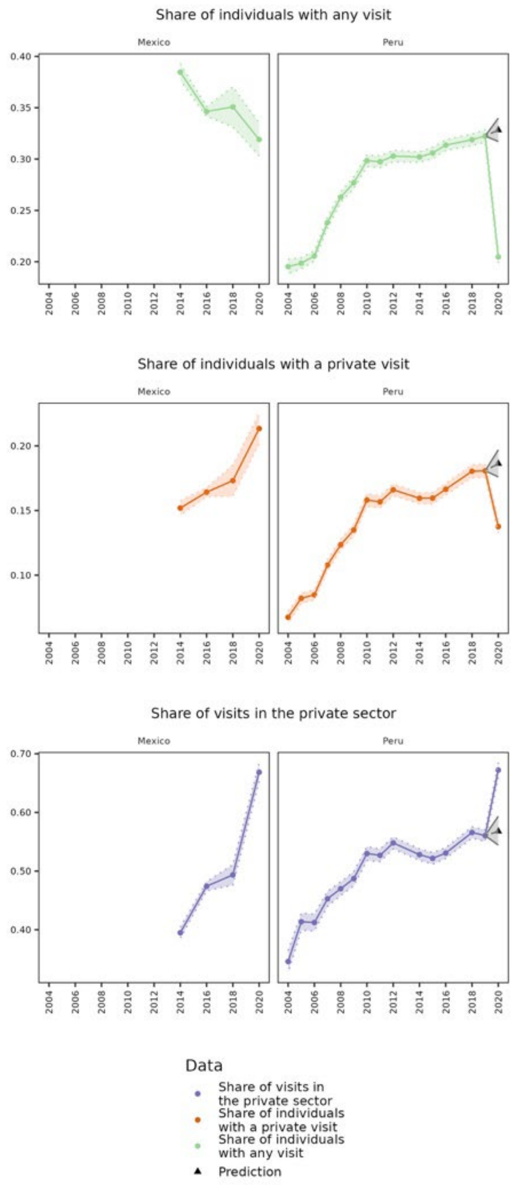

Note: Predictions were not possible for Mexico due to lack of historical data.

Section 5. Interrupted time series analysis of stay-at-home-orders

Our interrupted time series (ITS) approach was used to conduct two separate analyses: one for the healthcare visits per person variable and another for the private share of healthcare visits outcome. In both, we are testing for a shift in the level and trend of the outcome at the time both countries implemented stay-at-home orders in response to the global COVID-19 pandemic. We used distinct transformations of the outcome and specifications for each. Because we have different time frames of data available for the respective countries, slightly different specifications are used in each.

For the following equations, Y_t represents utilization rates or private sector share in 2018, 2019 or 2020 by month; $COVID$ is an indicator representing April through December 2020; $COVID * month$ is an interaction between the COVID period and the month of year count. We are interested in β_3, the change in levels when lockdowns commenced, and β_4, the shift in the monthly trends when lockdowns commenced.

Private share visits. To examine the share of healthcare visits in the private sector, we use a simple ITS approach, but we use a slightly different specification in each of the countries.

In Peru, we have a continuous series, spanning 2019-2020. We thus specified the following OLS regression for private share visits in Peru:

$$Y_t = \beta_1 + \beta_2 month + \beta_3 COVID + \beta_4 COVID * month + \varepsilon_t$$

Where $month$ is a count for month in the series (1-24).

In Mexico, we do not have a continuous series, but instead have data from 2018 to make a stronger estimate of the pre-pandemic trend. For private share visits in Mexico, we estimated the following regression:

$$Y_t = \beta_1 + \beta_2 month + \beta_3 COVID + \beta_4 COVID * month + \varepsilon_t + \beta_5 Year_{2020}$$

Where $month$ is a count for month of the year (1-12) and $Year_{2020}$ is an indicator representing the year 2020. The intercept for 2020 ensures that $\beta_3$and β_4 are not estimating the change in levels between 2018 and 2020.

Healthcare visits per person. In each of the countries, we used a differenced ITS approach for both all healthcare visits per person and private healthcare visits per person. A differenced approach was required because there is substantial seasonal variation in healthcare visits per person, part of which is due to the method of data collection (recall periods and period of data collection) used in each of the countries. In Peru, we thus divided the healthcare visits per month by the prior year's healthcare visits per month (e.g., January 2020 divided by January 2019). In Mexico, we use the same approach but divide by 2018 rather than 2019, since the most recent year of data is available for 2018 only. We have no reason to believe that seasonal patterns would be substantially different in 2019 versus 2018 in Mexico, but it does affect the interpretation of the results.

Because we have a similar transformation of data and are using the prior years in similar ways, we use the same specification in both Peru and Mexico:

$$Y_t = \beta_1 + \beta_2 month + \beta_3 COVID + \beta_4 COVID * month + \varepsilon_t$$

Where $month$ is a count for month of the year (1-12), although we are only able to extend the analysis until September 2020 in Mexico. Because data collection spanned August through November, once we extend beyond the midpoint of data collection, we encounter substantial differences due to the varying samples (e.g. data collection did not proceed exactly the same in 2020 versus 2018 – different regions and logistics made the samples distinct, making the results of the differenced healthcare visits per person inconsistent).

We provide the full set of results for these ITS regressions in the two supplementary tables below.

Supplementary table 9a: Interrupted time series regressions, Mexico

	Mexico					
	Private healthcare visits relative to prior year (2018), by month (n=9)		Healthcare visits relative to prior year (2018), by month (n=9)		Private sector share of healthcare, by month (n=22)	
	Coefficient and standard error	Statistical significance	Coefficient and standard error	Statistical significance	Coefficient and standard error	Statistical significance
Month	-0.007	0.875	-0.006	0.247	0.006	0.001
	(0.004)		(0.001)		(0.0002)	
COVID-19 Period	0.124	0.329	-0.061	0.009	0.070	0.006
	(0.1396)		(0.003)		(0.040)	
Month * COVID-19 Period	-0.008	0.870	0.005	0.331	-0.001	0.815
	(0.029)		(0.001)		(0.005)	
Year 2020	-	-	-	-	0.134	<.001
	-		-		(0.004)	

Supplementary table 9b: Interrupted time series regressions, Peru

	Peru					
	Private healthcare visits relative to prior year (2019), by month (n=12)		Healthcare visits relative to prior year (2019), by month (n=12)		Private sector share of healthcare, by month (n=24)	
	Coefficient and standard error	Statistical significance	Coefficient and standard error	Statistical significance	Coefficient and standard error	Statistical significance
Month	-0.074	0.007	-0.101	0.001	-0.0003	0.813
	(0.004)		(<0.001)		(0.001)	
COVID-19 Period	-0.700	<.001	-0.925	<.001	0.336	<.001
	(0.023)		(0.022)		(0.054)	
Month * COVID-19 Period	0.116	<.001	0.136	<.001	-0.008	0.012
	(0.005)		(0.003)		(0.002)	

Section 6. Analysis of cross-sectional variation in COVID-19 outcomes

Section 6a. COVID-19 and healthcare expenditure and utilization

We conducted an ecological spatial analysis to assess the association between CHE and its drivers and the burden of COVID-19 at the administrative division of residence in Mexico and Peru. We hypothesized that CHE, OOP expenditure and healthcare use might increase where there are more COVID-19 deaths if COVID-19 care was *additional to* other healthcare. Alternatively, they might decline where there are more COVID-19 deaths if potential healthcare users stayed away from health facilities – a situation where COVID-19 care may have *displaced* or *repelled* other healthcare users. We restricted our analysis to administrative divisions with responses from at least 20 households but conduct sensitivity analyses with other thresholds (shown in the following section). All variables produced from the microdata were averaged by the administrative unit in which the cluster was located using survey weights and then merged with COVID-19 information that was available only at the administrative unit level. Not all administrative units are captured in the analysis because of the sampling in the surveys. Where there are data, there are only one-to-one matches however – we do not conduct a multilevel analysis.

We estimated the association between CHE and its drivers at the subnational level with log-transformed COVID-19 deaths per 100,000 population ($COVID19$) for each municipality in Mexico and each district in Peru (i), controlling for: log-transformed average consumption expenditure per household member ($\log\ consumption\ expenditure\ pp_i$), the proportion of people older than 25 with less than post-secondary education ($secondary\ educ\ prop_i$), average age (age_i), and the proportion of people living in a rural area ($rural\ prop_i$), with the following:

$$Y_i = \beta_1 + \beta_2 \log COVID19\ deaths_i + \beta_3 \log\ consumption\ expenditure\ pp_i + \beta_4 secondary\ educ\ prop_i + \beta_5 age_i + \beta_6 rural\ prop_i + \varepsilon_i \quad (1)$$

Where Y_i is: CHE (10%); CHE (25%); log-transformed average OOP spending per household member; share of respondents using any healthcare; share of respondents using private health care; and the share of healthcare visits that took place in the private sector.

In Mexico, where municipality of residence (n = 2,388) was available for each household, we matched household clusters with Ministry of Health data on COVID-19 hospitalization, case, and death rates by municipality.[11] In Peru, where district of residence (n = 1,720) was made available for each household, we matched household cluster data with COVID-19 hospitalization, case, and death rates by district with Ministry of Health data.[12]

Section 6b. COVID-19 data sources

We obtained data on COVID-19 metrics from the Secretariat of Health's Historical Open Database 2020 for Mexico and the Ministry of Health's Open Database for Peru on 09/30/2021.[12,13] From these data sources, we extracted information on the number of COVID-19 tests, cases, hospitalizations, and deaths. In Peru, COVID-19 metrics were reported by health systems and collected by the National Institute of Health (INS) and the National Center for Epidemiology, Prevention and Disease Control (MINSA). Similarly, health systems reported COVID-19 metrics and the General Directorate of Epidemiology collected these metrics in Mexico.

For this analysis, test counts describe all patients who received a COVID-19 test where the laboratory reported the results. Data describing the number of tests was only available for Mexico. Cases consist of the daily record of positive cases of COVID-19 confirmed with any type of test where the patient presented symptoms. Hospitalizations include COVID-19 cases that were admitted to the hospital. Deaths correspond to the total number of people who died in a healthcare facility and tested positive for COVID-19. COVID-19 metrics between April and December in 2020 were aggregated to the district level in Peru; COVID-19 metrics from April through November, the end of ENIGH survey collection period, were aggregated to the municipality level in Mexico.

Rates were calculated using the most recent publicly available population data at the municipality and district levels for Mexico and Peru respectively. Population counts for municipalities in 2020 were extracted from the Census of Population and Housing 2020 conducted by National Institute of Statistics and Geography (INEGI) for Mexico; estimated population counts for districts in 2020 were based off the 2017 Peru Population and Housing Census conducted by National Institute of Statistics and Informatics (INEI)[14].

There are different recall periods and information on timing available in the two countries' surveys, which resulted in us merging on slightly different time frames in each country. The recall period was 4 weeks for household consumption expenditure, household OOP health spending and CHE (10% and 25%), and 1 year for healthcare utilization in Mexico. The recall period was 4 weeks for household consumption expenditure, household OOP health spending and CHE (10% and 25%), and healthcare utilization in Peru. Spending and CHE measures were annualized for this analysis. To be align with these recall periods after adjustment using the available COVID-19 data, we merged these covariates on cumulative COVID-19 metrics between April and November in Mexico, and between April and December in Peru.

We employed a trimming approach based on household counts by geography to stabilize regression results. We opted to use municipalities and districts with 20 or more households in the final analyses because it permitted us to use the most expansive portion of the data possible (96.8% or 86,154 households in 910 municipalities in Mexico and 71.1% of available observations or 18,459 households in 331 districts in Peru). We present sensitivity analyses that use varying levels of this cutoff in the following section 6e.

Section 6c. Model specifications

Predictor and outcome measures in the COVID-19 regression analyses were calculated for each respondent or household using microdata from Mexico and Peru and then aggregated to each cluster. We calculated household OOP health spending per household member, catastrophic health expenditure (10% and 25%), household consumption per household member, and rurality were measured at the household level. Age and indicators of any healthcare visit and private healthcare visit were measured among all respondents. Post-secondary educational attainment was measured among respondents over 25 and share of healthcare visits in the private sector was

measured among respondents with a healthcare visit in the past year in Mexico or the past four weeks in Peru. All predictor and outcome measures were aggregated to the municipality and district levels for Mexico and Peru respectively using survey weights. Covariates were selected based on existing research and theory.

Section 6d. Supplementary results

In Figure 9, we depict the coefficients from the regressions of CHE and its drivers on sub-national variation in the natural log-transformed COVID-19 death rate. We opted to focus on the COVID-19 mortality analysis as our main results due to concerns about missingness in the other COVID-19 metrics. While low case and hospitalizations rates may reflect insufficient testing resources in a municipality or district, we believe that existing national health information systems in Mexico and Peru are better able to capture COVID-19 deaths, although there is likely to be some missingness in these data sources as well.[15] We found that, in Mexico, where the COVID-19 death rate was higher, OOP health spending, CHE (10%), CHE (25%), and private sector visits were lower. In Peru, in contrast, we find that OOP health spending, CHE (10%), CHE (25%), healthcare use, and private sector healthcare use were all higher where COVID-19 deaths were higher. Full regression results are in tables 10-17.

In interpreting these results, it is important to note the overall change in healthcare use in the two countries. While healthcare use dropped substantially overall in Peru, the cross-sectional analysis indicates that it fell less in areas where COVID-19 mortality was higher – suggesting that people may have been more likely to leave home for COVID-19 care for other types of care during this period. Strict stay at home orders may have also prevented people from getting care that, in Mexico, was still being pursued. In Mexico, health care use did not drop as substantially. Thus, the finding that healthcare use was lower in areas with higher COVID-19 death rates suggests that people may have put off care in areas where health facilities were treating many COVID-19 patients but were not deterred from health care use in areas where there were fewer deaths from COVID-19, as they may have in Peru.

Supplementary figure 9. Association between CHE and CHE drivers with subnational variation in the log COVID-19 death rate in Mexico and Peru, 2020

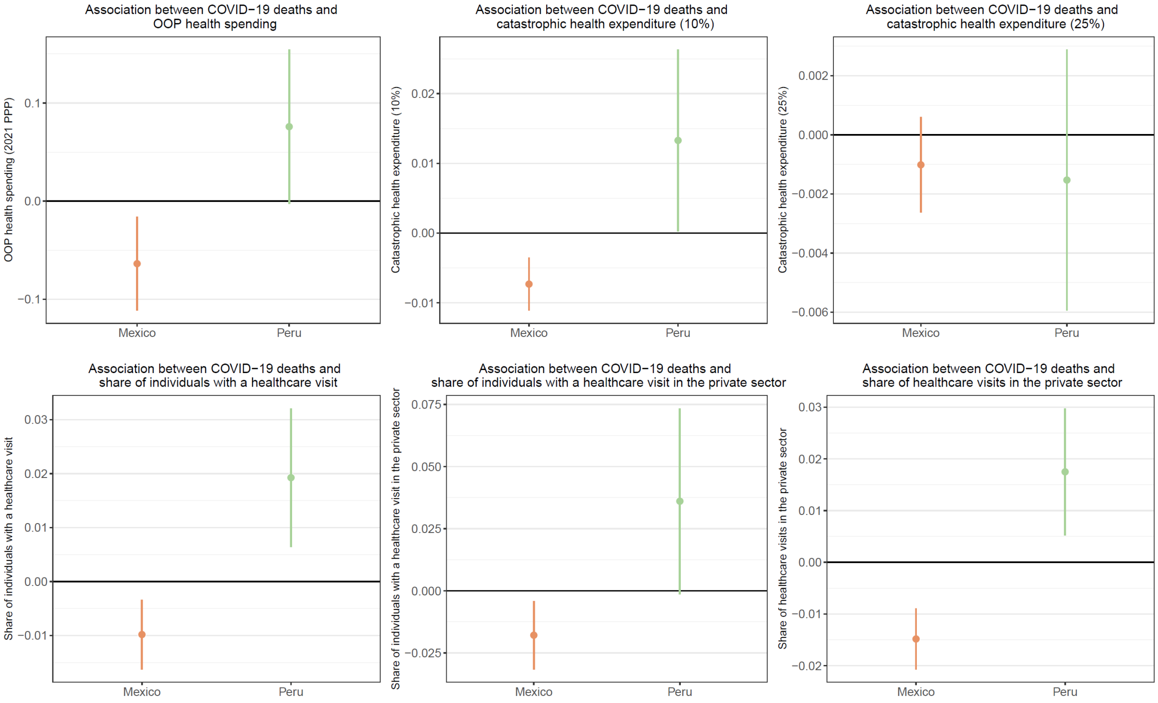

Notes: The point in each plot represents the coefficient estimate and the line surrounding the point represents the 95% uncertainty interval surrounding the coefficient estimate.

Supplementary tables 10-17 provide results of the models estimating the associations with outcomes and each of the COVID-19 metrics.

Supplementary table 10. Linear probability model of healthcare expenditure and utilization on COVID-19 deaths

	Household OOP health spending per household member	Catastrophic health expenditure (10%)	Catastrophic health expenditure (25%)	Share of individuals with a healthcare visit	Share of individuals with a private healthcare visit	Share of healthcare visits in the private sector
**Mexico**						
Logged death rate per 100,000	-0.0449* (0.02)	-0.00435* (0.00184)	-0.00184* (0.000761)	-0.00510 (0.00274)	-0.0100*** (0.00246)	-0.0160** (0.00585)
Logged household consumption per household member	1.27 (0.0833)	0.0212 (0.00767)	0.0156 (0.00317)	0.0125 (0.0114)	0.0155 (0.0103)	0.0655 (0.0244)
Share of individuals over 25 with less than post-secondary education	0.497 (0.187)	0.0361 (0.0172)	0.0242 (0.00712)	0.0146 (0.0256)	0.0136 (0.023)	-0.0182 (0.0548)
Average age	0.0205 (0.00641)	0.00302 (0.00059)	0.000308 (0.000244)	0.000459 (0.000878)	-0.00107 (0.000789)	-0.00489 (0.00188)
Share rural	-0.0667 (0.057)	-0.00144 (0.00524)	0.00264 (0.00217)	-0.00405 (0.0078)	-0.0224 (0.00701)	-0.106 (0.0167)
**Peru**						
Logged death rate per 100,000	0.0796* (0.0324)	0.0136** (0.00515)	0.000573 (0.00191)	0.0166** (0.00522)	0.0176*** (0.00477)	0.048** (0.015)
Logged household consumption per household member	1.17 (0.104)	0.0265 (0.0166)	0.00232 (0.00613)	0.0216 (0.0195)	0.027 (0.0178)	0.113 (0.0561)
Share of individuals over 25 with less than post-secondary education	0.582 (0.231)	0.0649 (0.0369)	0.0262 (0.0137)	0.115 (0.044)	0.268 (0.127)	0.118 (0.0403)
Average age	-0.00241 (0.00587)	0.000909 (0.000935)	0.000416 (0.000346)	0.000662 (0.00111)	0.00128 (0.00102)	0.00276 (0.0032)
Share rural	-0.0599 (0.0973)	0.00301 (0.0155)	-0.00133 (0.00574)	0.000662 (0.00111)	-0.0481 (0.0167)	-0.2 (0.0525)

Supplementary table 10 describes coefficient estimate and standard error. Statistical significance is notated as follows: * p < 0.05; ** p < 0.01 ; *** p , 0.001.

Supplementary table 11. Binomial model of healthcare expenditure and utilization on COVID-19 deaths

	**Household OOP health spending per household member**	**Catastrophic health expenditure (10%)**	**Catastrophic health expenditure (25%)**	**Share of individuals with a healthcare visit**	**Share of individuals with a private healthcare visit**	**Share of healthcare visits in the private sector**
**Mexico**						
Logged death rate per 100,000	-0.0368* (0.019)	0.886*** (1.02)	0.924* (1.04)	0.974*** (1.01)	0.943*** (1.01)	0.973*** (1.01)
Logged household consumption per household member	1.28*** (0.0749)	1.18* (1.07)	1.59*** (1.15)	1* (1.02)	0.985* (1.02)	0.999* (1.03)
Share of individuals over 25 with less than post-secondary education	0.465** (0.173)	1.64** (1.19)	3.35*** (1.39)	1.01* (1.05)	0.866* (1.06)	0.884* (1.07)
Average age	0.0208*** (0.00599)	1.06*** (1.01)	1.04*** (1.01)	1* (1)	0.994** (1)	0.993** (1)
Share rural	-0.105* (0.0554)	0.954* (1.06)	1.01* (1.13)	0.985* (1.02)	0.796*** (1.02)	0.84*** (1.02)
**Peru**						
Logged death rate per 100,000	0.0796* (0.0324)	1.11* (1.04)	1.17* (1.11)	1.09*** (1.02)	1.11*** (1.02)	1.03* (1.02)
Logged household consumption per household member	1.17*** (0.104)	1.5** (1.15)	0.935* (1.43)	1.23*** (1.06)	1.29*** (1.07)	1.06* (1.09)
Share of individuals over 25 with less than post-secondary education	0.582* (0.231)	1.79* (1.37)	1.89* (2.18)	2.3*** (1.14)	2.94*** (1.16)	1.3* (1.2)
Average age	-0.00241* (0.00587)	0.995* (1.01)	1.02* (1.02)	1.01*** (1)	1.03*** (1)	1.02** (1.01)
Share rural	-0.0599* (0.0973)	0.931* (1.16)	1.3* (1.41)	0.95* (1.06)	0.605*** (1.07)	0.663*** (1.09)

Supplementary table 11 describes coefficient estimate and standard error. Statistical significance is notated as follows: * p < 0.05; ** p < 0.01 ; *** p , 0.001.

Supplementary table 12. Linear probability model of healthcare expenditure and utilization on COVID-19 hospitalizations

	Household OOP health spending	Catastrophic health expenditure (10%)	Catastrophic health expenditure (25%)	Share of individuals with a healthcare visit	Share of individuals with a private healthcare visit	Share of healthcare visits in the private sector
**Mexico**						
Logged hospitalization rate per 100,000	-0.0668** (0.0237)	-0.00539* (0.00219)	-0.0022* (0.000905)	-0.00816* (0.00325)	-0.013*** (0.00292)	-0.02** (0.00697)
Logged household consumption per household member	1.29*** (0.0843)	0.0224** (0.00776)	0.016*** (0.00321)	0.0156 (0.0115)	0.0189 (0.0104)	0.0701** (0.0247)
Share of individuals over 25 with less than post-secondary education	0.479* (0.187)	0.0353* (0.0172)	0.024*** (0.00713)	0.0121 (0.0256)	0.0114 (0.023)	-0.0211 (0.0548)
Average age	0.0202** (0.0064)	0.00299*** (0.00059)	0.000298 (0.000244)	0.000429 (0.000877)	-0.00113 (0.000788)	-0.00497** (0.00188)
Share rural	-0.0641 (0.0568)	-0.00109 (0.00523)	0.0028 (0.00217)	-0.00381 (0.00778)	-0.0216** (0.00698)	-0.105*** (0.0167)
**Peru**						
Logged hospitalization rate per 100,000	-0.00233 (0.0181)	0.00404 (0.00288)	-0.000922 (0.00106)	0.00436 (0.00319)	0.00494 (0.00293)	0.00453 (0.0092)
Logged household consumption per household member	-0.00233 (0.0181)	0.0311 (0.0166)	0.00327 (0.00608	0.0237 (0.0198)	0.0291 (0.0181)	0.124* (0.0571)
Share of individuals over 25 with less than post-secondary education	0.543* (0.233)	0.0565 (0.0371)	0.0263 (0.0136)	0.0972* (0.0443)	0.0984* (0.0407)	0.219 (0.128)
Average age	-0.00284 (0.00593)	0.0565 (0.0371)	0.0263 (0.0136)	0.0972* (0.0443)	0.00151 (0.00104)	0.00319 (0.00326)
Share rural	-0.139 (0.0928)	-0.00964 (0.0148)	-0.00208 (0.00542)	-0.0324 (0.0177)	-0.0641*** (0.0162)	-0.248*** (0.0511)

Supplementary table 12 describes coefficient estimate and standard error. Statistical significance is notated as follows: * p < 0.05; ** p < 0.01 ; *** p , 0.001.

Supplementary table 13. Binomial model of healthcare expenditure and utilization on COVID-19 hospitalizations

	**Household OOP health spending**	**Catastrophic health expenditure (10%)**	**Catastrophic health expenditure (25%)**	**Share of individuals with a healthcare visit**	**Share of individuals with a private healthcare visit**	**Share of healthcare visits in the private sector**
**Mexico**						
Logged hospitalization rate per 100,000	-0.05* (0.0229)	0.87*** (1.02)	0.917* (1.04)	0.968*** (1.01)	0.937*** (1.01)	0.972** (1.01)
Logged household consumption per household member	1.29*** (0.076)	1.2* (1.08)	1.6*** (1.15)	1.00* (1.02)	0.989* (1.02)	0.998* (1.03)
Share of individuals over 25 with less than post-secondary education	0.456** (0.173)	1.6** (1.19)	3.31*** (1.39)	0.998* (1.05)	0.855** (1.06)	0.881* (1.07)
Average age	0.0205*** (0.00598)	1.06*** (1.01)	1.04*** (1.01)	1.00* (1)	0.993** (1)	0.993** (1)
Share rural	-0.103* (0.0552)	0.968* (1.06)	1.02* (1.13)	0.987* (1.02)	0.801*** (1.02)	0.843*** (1.02)
**Peru**						
Logged hospitalization rate per 100,000	-0.00233* (0.0181)	1.02* (1.02)	1.01* (1.05)	1.03*** (1.01)	1.05*** (1.01)	1.01* (1.01)
Logged household consumption per household member	1.22*** (0.104)	1.59*** (1.15)	1.04 (1.42)	1.24*** (1.06)	1.3*** (1.07)	1.07 (1.09)
Share of individuals over 25 with less than post-secondary education	0.543* (0.233)	1.76* (1.37)	1.82* (2.18)	2.2*** (1.14)	2.81*** (1.16)	1.29* (1.2)
Average age	-0.00284* (0.00593)	0.995* (1.01)	1.02* (1.02)	1.02*** (1)	1.03*** (1)	1.02** (1.01)
Share rural	-0.139* (0.0928)	0.825* (1.15)	1.11* (1.38)	0.871* (1.06)	0.538*** (1.07)	0.647*** (1.08)

Supplementary table 13 describes coefficient estimate and standard error. Statistical significance is notated as follows: * p < 0.05; ** p < 0.01 ; *** p , 0.001.

Supplementary table 14. Linear probability model of healthcare expenditure and utilization on COVID-19 cases

	Household OOP health spending	Catastrophic health expenditure (10%)	Catastrophic health expenditure (25%)	Share of individuals with a healthcare visit	Share of individuals with a private healthcare visit	Share of healthcare visits in the private sector
**Mexico**						
Logged case rate per 100,000	-0.0868***<br>(0.0215)	-0.00839***<br>(0.00197)	-0.0022**<br>(0.000821)	-0.0125***<br>(0.00293)	-0.0188***<br>(0.0026)	-0.0304***<br>(0.00627)
Logged household consumption per household member	1.32***<br>(0.0838)	0.0258***<br>(0.0077)	0.0162***<br>(0.00321)	0.0207<br>(0.0115)	0.0258*<br>(0.0102)	0.0822***<br>(0.0245)
Share of individuals over 25 with less than post-secondary education	0.429*<br>(0.187)	0.429*<br>(0.187)	0.0232**<br>(0.00715)	0.00347<br>(0.0255)	0.00347<br>(0.0255)	-0.0418<br>(0.0546)
Average age	0.0212***<br>(0.00638)	0.0212***<br>(0.00638)	0.000324<br>(0.000244)	0.000568<br>(0.000872)	-0.000916<br>(0.000774)	-0.000916<br>(0.000774)
Share rural	-0.0665<br>(0.0565)	-0.00142<br>(0.0052)	0.00279<br>(0.00216	-0.00429<br>(0.00772)	-0.0223**<br>(0.00686)	-0.106***<br>(0.0165)
**Peru**						
Logged case rate per 100,000	0.0659<br>(0.0345)	0.0082<br>(0.00551)	-0.00162<br>(0.00202)	0.0172**<br>(0.00608)	0.00921<br>(0.00564)	-0.00895<br>(0.0177)
Logged household consumption per household member	1.18***<br>(0.105)	0.0294<br>(0.0167)	0.00351<br>(0.00614)	0.0207<br>(0.0196)	0.0291<br>(0.0181)	0.13<br>(0.0571)
Share of individuals over 25 with less than post-secondary education	0.61**<br>(0.234)	0.0665<br>(0.0375)	0.0243<br>(0.0138)	0.122**<br>(0.0446)	0.113**<br>(0.0413)	0.21*<br>(0.13)
Average age	-0.00134<br>(0.00594)	0.00102<br>(0.000949)	0.000377<br>(0.000349)	0.00103<br>(0.00112)	0.00155<br>(0.00104)	0.00296<br>(0.00326)
Share rural	-0.108<br>(0.0936)	-0.00668<br>(0.015)	-0.00264<br>(0.00549)	-0.0249<br>(0.0178)	-0.0612**<br>(0.0165)	-0.254***<br>(0.0518)

Supplementary table 14 describes coefficient estimate and standard error. Statistical significance is notated as follows: * $p < 0.05$; ** $p < 0.01$; *** p , 0.001.

Supplementary table 15. Binomial model of healthcare expenditure and utilization on COVID-19 cases

	Household OOP health spending	Catastrophic health expenditure (10%)	Catastrophic health expenditure (25%)	Share of individuals with a healthcare visit	Share of individuals with a private healthcare visit	Share of healthcare visits in the private sector
**Mexico**						
Logged case rate per 100,000	-0.0629** (0.0208)	0.847*** (1.02)	0.882*** (1.04)	0.95*** (1.01)	0.905*** (1.01)	0.958*** (1.01)
Logged household consumption per household member	1.31*** (0.0753)	1.24** (1.08)	1.68*** (1.15)	1.03* (1.02)	1.03* (1.02)	1.02* (1.03)
Share of individuals over 25 with less than post-secondary education	0.41* (0.174)	1.39* (1.19)	2.93** (1.4)	0.946* (1.05)	0.769*** (1.06)	0.844* (1.07)
Average age	0.021*** (0.00597)	1.06*** (1.01)	1.04*** (1.01)	1* (1)	0.994** (1)	0.993** (1)
Share rural	-0.106* (0.0551)	0.967* (1.06)	1.02* (1.13)	0.985* (1.02)	0.799*** (1.02)	0.842*** (1.02)
**Peru**						
Logged case rate per 100,000	0.0659* (0.0345)	1.01* (1.05)	1.05* (1.12)	1.12*** (1.02)	0.963* (1.02)	0.963* (1.02)
Logged household consumption per household member	1.18*** (0.105)	1.6*** (1.15)	1.02* (1.42)	1.23*** (1.06)	1.08* (1.09)	1.08* (1.09)
Share of individuals over 25 with less than post-secondary education	0.61** (0.234)	1.78* (1.37)	1.91* (2.2)	2.56*** (1.14)	1.22* (1.21)	1.22* (1.21)
Average age	-7.9596E-06	0.995* (1.01)	1.02* (1.02)	1.02*** (1)	1.02** (1.01)	1.02** (1.01)
Share rural	-0.0101088	0.826* (1.15)	1.14* (1.39)	0.916* (1.06)	0.629*** (1.09)	0.629*** (1.09)

Supplementary table 15 describes coefficient estimate and standard error. Statistical significance is notated as follows: * p < 0.05; ** p < 0.01 ; *** p , 0.001.

Supplementary table 16. Linear probability model of healthcare expenditure and utilization on COVID-19 tests

	Household OOP health spending	Catastrophic health expenditure (10%)	Catastrophic health expenditure (25%)	Share of individuals with a healthcare visit	Share of individuals with a private healthcare visit	Share of healthcare visits in the private sector
**Mexico**						
Logged test rate per 100,000	-0.095*** (0.0222)	-0.01*** (0.00204)	-0.00256** (0.000851)	-0.0128*** (0.00304)	-0.0128*** (0.00304)	-0.0299*** (0.00651)
Logged household consumption per household member	1.3*** (0.0824)	0.0246** (0.00756)	0.0158*** (0.00316)	0.0174 (0.0113)	0.0205* (0.01)	0.0731** (0.0241)
Share of individuals over 25 with less than post-secondary education	0.384* (0.188)	0.0238 (0.0173)	0.0218** (0.0072)	-0.00156 (0.0258)	-0.00775 (0.0229)	-0.0519 (0.0551)
Average age	-0.0519 (0.0551)	0.00304*** (0.000584)	0.000312 (0.000244)	0.000494 (0.000871)	-0.00103 (0.000775)	-0.00482** (0.00187)
Share rural	-0.0617 (0.0564)	-0.000988 (0.00517)	0.0029 (0.00216)	-0.00356 (0.00772)	-0.0211** (0.00687)	-0.104*** (0.0165)

Supplementary table 16 describes coefficient estimate and standard error. Statistical significance is notated as follows: * p < 0.05; ** p < 0.01 ; *** p , 0.001. Data on test rates was only available for Mexico.

Supplementary table 17. Binomial model of healthcare expenditure and utilization on COVID-19 tests

Mexico						
	**Household OOP health spending**	**Catastrophic health expenditure (10%)**	**Catastrophic health expenditure (25%)**	**Share of individuals with a healthcare visit**	**Share of individuals with a private healthcare visit**	**Share of healthcare visits in the private sector**
Logged test rate per 100,000	-0.0664** (0.0217)	0.849*** (1.02)	0.871*** (1.04)	0.947*** (1.01)	0.906*** (1.01)	0.959*** (1.01)
Logged household consumption per household member	1.29*** (0.074)	1.18* (1.07)	1.63*** (1.15)	1.01* (1.02)	1.00* (1.02)	1.00* (1.03)
Share of individuals over 25 with less than post-secondary education	0.389* (0.175)	1.26* (1.19)	2.64** (1.4)	0.911* (1.05)	0.724*** (1.06)	0.826** (1.07)
Average age	0.0207*** (0.00597)	1.06*** (1.01)	1.04*** (1.01)	1* (1)	0.993*** (1)	0.993** (1)
Share rural	-0.0057304	0.987* (1.06)	1.03* (1.13)	0.991* (1.02)	0.809*** (1.02)	0.846*** (1.02)

Supplementary table 17 describes coefficient estimate and standard error. Statistical significance is notated as follows: * p < 0.05; ** p < 0.01 ; *** p , 0.001. Data on test rates was only available for Mexico.

Section 6e. Sensitivity analyses

In this section, we present results from varying the cutoff of the number of households per cluster in the linear probability model. We depict the regression coefficients and p-values from regressions using different cutoffs for minimum number of households per administrative unit. The direction of the coefficient for our covariate of interest, death rates, does not differ for all dependent variables with the exception of CHE (25%) in Peru. The cutoff does tend to have an influence on the statistical significance of the results. As fewer units are available – by enforcing a higher cutoff – the results tend to no longer be statistically significant. Since the direction of the results stay the same regardless of the cutoff in almost all cases, we interpret the cutoff value as giving us statistical power.

Supplementary figure 10. Coefficients from sensitivity analysis of COVID-19 mortality regressions using different cut-off thresholds

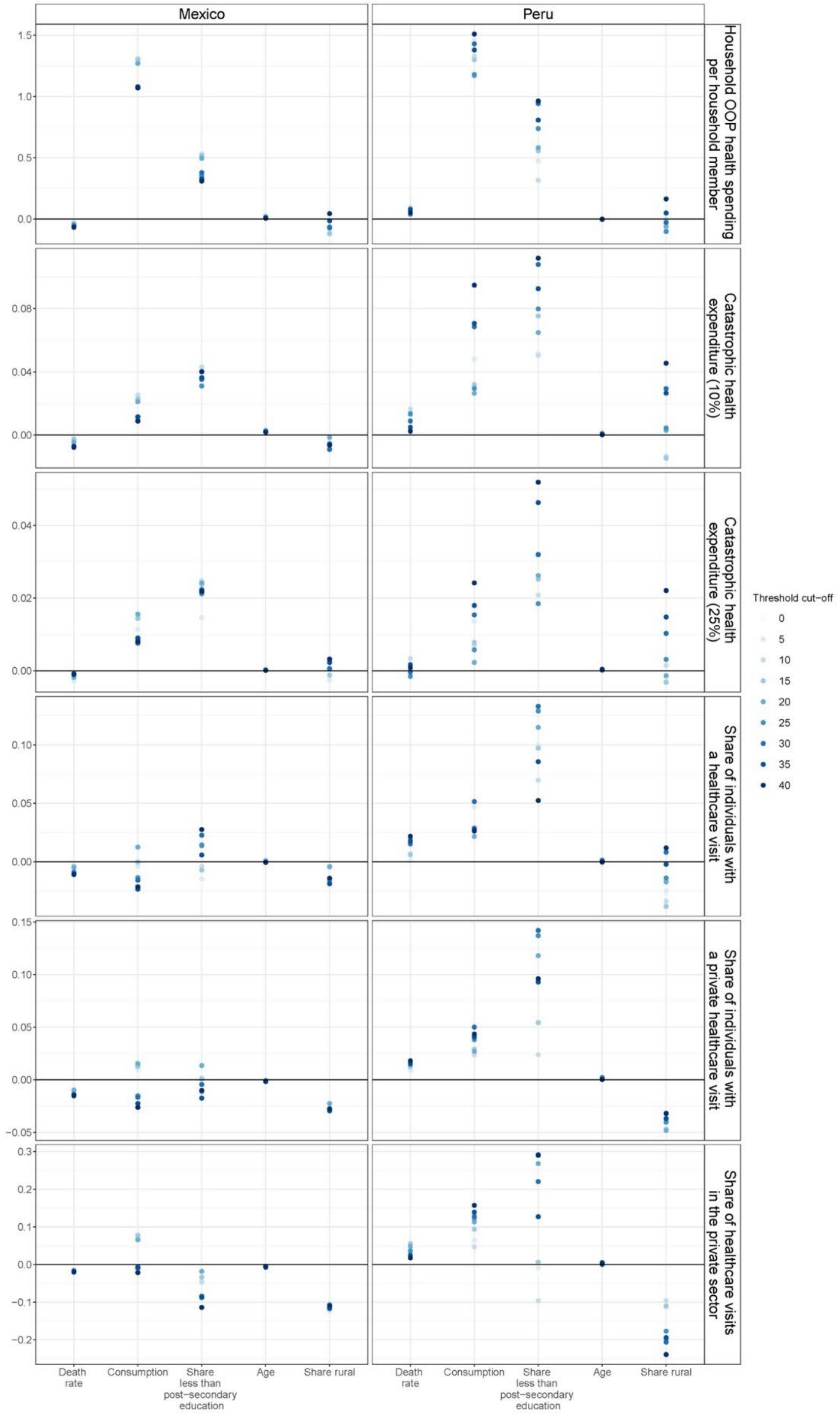

Supplementary figure 11. P-values from sensitivity analysis of COVID-19 mortality regressions using different cut-off thresholds

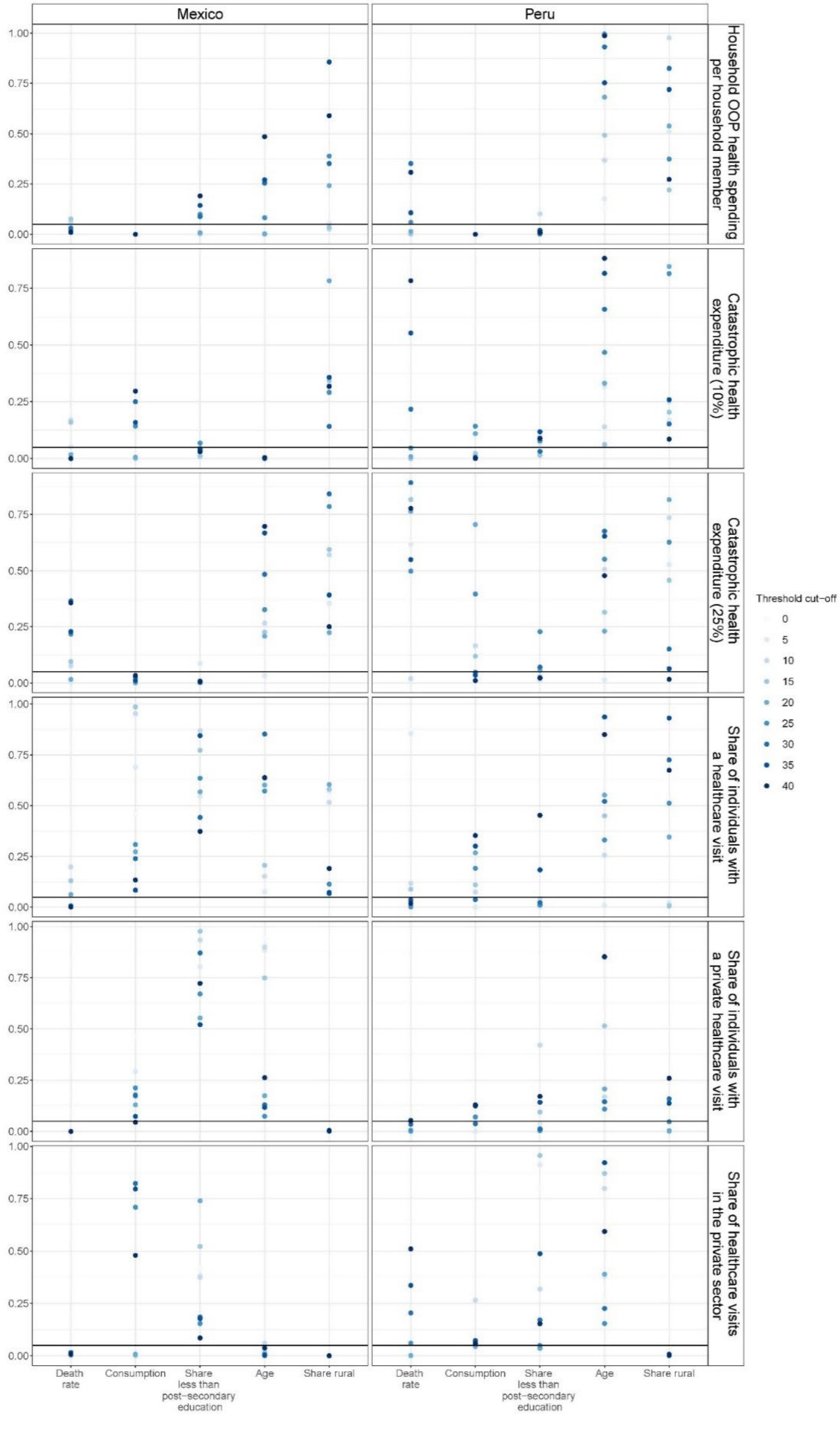

Part 2. Online tools

Data from the HEFPI database can be located here: https://datatopics.worldbank.org/health-equity-and-financial-protection/.

Part 3. List of abbreviations

CHE: Catastrophic Health Expenditure
HEFPI: Health Equity and Financial Protection Indicator database
FRP: Financial Risk Protection
OOP: Out-of-pocket

References

1 Stevens GA, Alkema L, Black RE, et al. Guidelines for Accurate and Transparent Health Estimates Reporting: the GATHER statement. The Lancet 2016; 388: e19–23.

2 Ritchie H, Roser M, Mispy J, Ortiz-Ospina E. SDG Indicators Metadata repository. 2022; published online Nov 1. https://unstats.un.org/sdgs/metadata/?Text=&Goal=3&Target=3.8 (accessed Jul 28, 2022).

3 National Institute of Statistics, Geography, and Informatics. ENIGH-84. National Survey of Household Income and Expenses. Methodological document. 1984. https://en.www.inegi.org.mx/app/biblioteca/ficha.html?upc=702825001746 (accessed Jul 28, 2023).

4 National Institute of Statistics and Informatics. Technical File: National Household Survey on Living Conditions and Poverty. 2020.

5 National Statistical Committee of the Republic of Belarus. Sample Household Living Standards Survey. Belstat. https://www.belstat.gov.by/en/gosudarstvennye-statisticheskie-nablyudeniya/vyborochnye-obsledovaniya/vyborochnoe-obsledovanie-domashnih-hozyaistv/ (accessed Jul 28, 2023).

6 Department of Living Standards Statistics and Household Surveys of the Federal State Statistics Service (Rosstat). Sample Survey of Household Budgets. Rosstat: System of access to survey results. 2019; published online Dec 10. https://obdx.gks.ru/ (accessed Feb 24, 2022).

7 The World Bank. Vietnam - Household Living Standards Survey 2004. 2018; published online Sept 19. https://microdata.worldbank.org/index.php/catalog/2370 (accessed July 28, 2023).

8 World Health Organization, World Bank. Global Monitoring Report on Financial Protection in Health 2021. Washington, DC: World Bank, 2021 https://openknowledge.worldbank.org/handle/10986/36723 (accessed Jan 13, 2022).

9 Peru National Household Survey on Living Conditions and Poverty 2020. 2021.

10 Encuesta Nacional de Ingresos y Gastos de los Hogares (ENIGH). 2020 Nueva serie. https://www.inegi.org.mx/programas/enigh/nc/2020/#Microdatos (accessed Dec 10, 2021).

11 Health Secretary. Open Data General Directorate of Epidemiology. gob.mx. 2022; published online Feb 24. http://www.gob.mx/salud/documentos/datos-abiertos-152127 (accessed Feb 24, 2022).

12 Peru Ministry of Health. Ministry of Health COVID-19 Open Data and Knowledge Management. Ministry of Health. https://www.minsa.gob.pe/datosabiertos/ (accessed Feb 24, 2022).

13 Salud S de. Datos Abiertos Dirección General de Epidemiología. gob.mx. http://www.gob.mx/salud/documentos/datos-abiertos-152127 (accessed Dec 10, 2021).

14 Instituto Nacional de Estadística e Informática. Perú: Proyecciones de Población Total según Departamento, Provincia y Distrito, 2018-2022. https://www.gob.pe/institucion/inei/informes-publicaciones/3464927-peru-proyecciones-de-poblacion-total-segun-departamento-provincia-y-distrito-2018-2022 (accessed Feb 24, 2023).

15 Total COVID-19 tests per 1,000 people. Our World in Data. https://ourworldindata.org/grapher/full-list-cumulative-total-tests-per-thousand (accessed Dec 5, 2022).
